# A New Methodology for Incorporating Chiral Linkers into Stapled Peptides

**DOI:** 10.1002/cbic.201700075

**Published:** 2017-05-18

**Authors:** Juan C. Serrano, James Sipthorp, Wenshu Xu, Laura S. Itzhaki, Steven V. Ley

**Affiliations:** ^1^ Department of Chemistry University of Cambridge Lensfield Road Cambridge CB2 1EW UK; ^2^ Department of Pharmacology University of Cambridge Tennis Court Road Cambridge CB2 1PD UK

**Keywords:** chiral linkers, protein–protein interactions, solid-phase synthesis, stapled peptides, two-component stapling

## Abstract

Stapled peptides have arisen as a new class of chemical probe and potential therapeutic agents for modulating protein–protein interactions. Here, we report the first two‐component *i*,*i*+7 stapling methodology that makes use of two orthogonal, on‐resin stapling reactions to incorporate linkers bearing a chiral centre into a p53‐derived stapled peptide. Post‐stapling modifications to the chain were performed on‐resin and enabled rapid access to various peptide derivatives from a single staple. The stapled peptides have increased helicity, protease stability and in vitro binding affinities to MDM2 compared to the equivalent unstapled peptide. This approach can be used to generate a library of diverse stapled peptides with different properties starting from a single stapled peptide, with scope for much greater functional diversity than that provided by existing stapling methodologies.

Protein–protein interactions (PPIs) govern the majority of biological processes and, as such, have been implicated in the progression of many diseases.^1^ Modulation of PPIs has become a major strategy for therapeutic interventions and the study of human disease at the molecular level;^2^ however, the large featureless contact surfaces of most PPIs have made them difficult to target through traditional methods using small molecules.^3^ Additionally, the intracellular location of many therapeutic targets has made them inaccessible to many biologics, which have an inherent difficulty in crossing cellular membranes.^4^ Peptide mimetics offer a potential middle‐ground approach owing to their similarity to the binding protein sequence (and, thus, likely high affinity and specificity) and their relative ease of synthesis and development.^5^ However, linear peptides have poor proteolytic stability and low cell permeability, which can limit their use to solely extracellular targets.^5^, ^6^

Peptide stapling can confer α‐helicity on linear peptides by restricting their conformational flexibility. This is achieved by crosslinking (stapling) the side chains of two amino acid residues within the sequence so as to stabilise the desired α‐helical conformation. Increasing the helicity of the peptide causes the secondary structure to resemble that of the mimicked protein fragment when bound, thus reducing the entropic penalty upon binding to its target protein.^7^ Additionally, stapling confers dramatic proteolytic stability both in vitro and in vivo.^8^ Many stapled peptides also exhibit increased cell permeability, although this is not a ubiquitous feature of stapling.^4^, ^8^, ^9^ Stapled peptides have been shown to modulate a diverse range of PPIs both in vitro and in vivo.^6^, ^10^

Historically, stapling strategies have employed a direct one‐component linkage between two amino acid residues on the peptide sequence to form the staple and confer structural rigidity (Figure 1 A). However, modifications to increase cell permeability or impart other functionalities require alteration of the peptide sequence, which can interfere with the affinity of the peptide for its protein target. Two‐component stapling seeks to remedy this by incorporating a bifunctional small‐molecule linker between the two residues (Figure 1 B). The staple linker can then function to both hold the peptide in the desired secondary structure and to add functionality. As such, many stapled peptides with different properties can be generated from a single linear peptide sequence.

**Figure 1:**
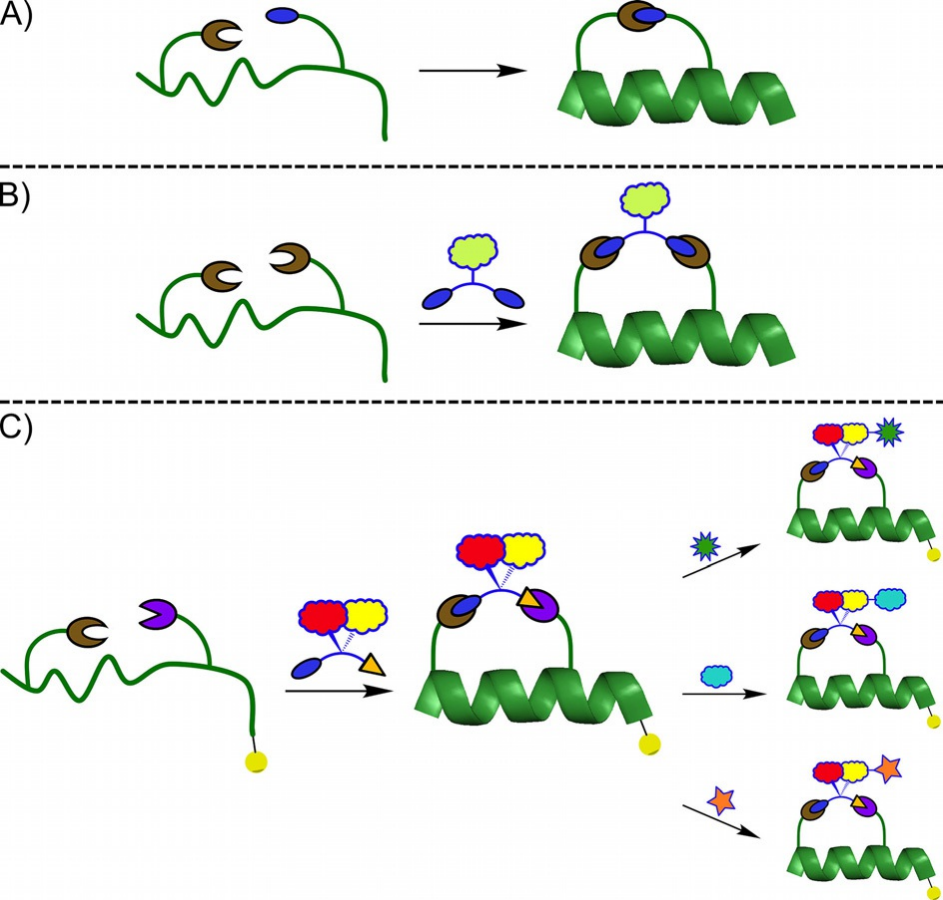
A) One‐component stapling. B) Two‐component, dual‐reaction stapling. C) Two‐component, sequential‐reaction stapling and expansion of the staple chain performed on‐resin to generate a diverse stapled‐peptide library from a single stapled peptide. The yellow ball represents the MBHA resin support.

Spring and co‐workers developed a two‐component *i,i*+7 double‐click stapling methodology by using a functional dialkyne linker that had been used to increase the permeability and binding affinity of both α‐helical and nonhelical peptides.^11^ However, performing azide‐alkyne cycloaddition on both stapling residues can limit the range of linkers that can be used to only symmetrical or achiral small molecules so as to avoid the formation of regioisomers or diastereomers. Additionally, as stapling is performed in solution on an unprotected peptide, further functionalisation is limited to bioorthogonal reactions, thereby reducing the scope of post‐stapling modifications. Thus, the generation of different staples involves the synthesis of separate linkers and subsequent formation of a new staple, requiring multiple iterations to generate a library of stapled peptides.

A more versatile two‐component stapling approach would allow the incorporation of asymmetric linkers. This strategy would not only increase the variety of appropriate linkers but also allow the effects of the stereochemistry of the staple chain on the biophysical properties and cellular activity of the peptide to be studied. The importance of the staple chain's stereochemistry was demonstrated by Li and co‐workers through incorporating a variable chiral centre into one‐component hydrocarbon stapled peptides.^12^ The separable diastereomers displayed significantly different helicities, cell permeability and protein‐target‐binding affinity based solely on the conformational difference of the staple chain. Previous studies have also shown that staple chain–protein binding occurs across various stapled peptide examples; this further justifies the use of the staple as a modification site for enhancing protein binding.^13^ Additionally, controlling the directional expansion of the staple chain has implications for the utility of post‐stapling modifications. Walensky and co‐workers have demonstrated that selective covalent targeting of BFL‐1 can be achieved by addition of an acrylamide moiety to the peptide sequence.^14^ We envisage that, in a similar manner, a staple chain could be expanded chemically to interact with surface cysteine residues on the target protein.

Herein, we report the development of an *i*,*i*+7, two‐component stapling methodology for incorporating linkers containing a chiral centre and enabling the isolation of a single diastereomer. This strategy uses two orthogonal reactions to allow the stepwise insertion of a bifunctional linker onto p53‐derived peptide **SP0** (Scheme 1 A). We chose ruthenium‐catalysed ring‐closing metathesis (RCM) and a copper catalysed azide–alkyne cycloaddition (CuAAC) as the stapling reactions owing to their chemoselectivity and widely reported use in peptide stapling.^15^ Both transformations were performed on‐resin by using **SP0** and linkers **1** and **2** to generate peptides **SP1** and **SP2** (Scheme 1 B and C). The pendant Fmoc‐protected lysine on linkers **1** and **2** was used as a reactive handle for further modification by solid‐phase peptide synthesis (SPPS) to attach additional amino acids or fluorescein isothiocyanate (FITC).

**Scheme 1:**
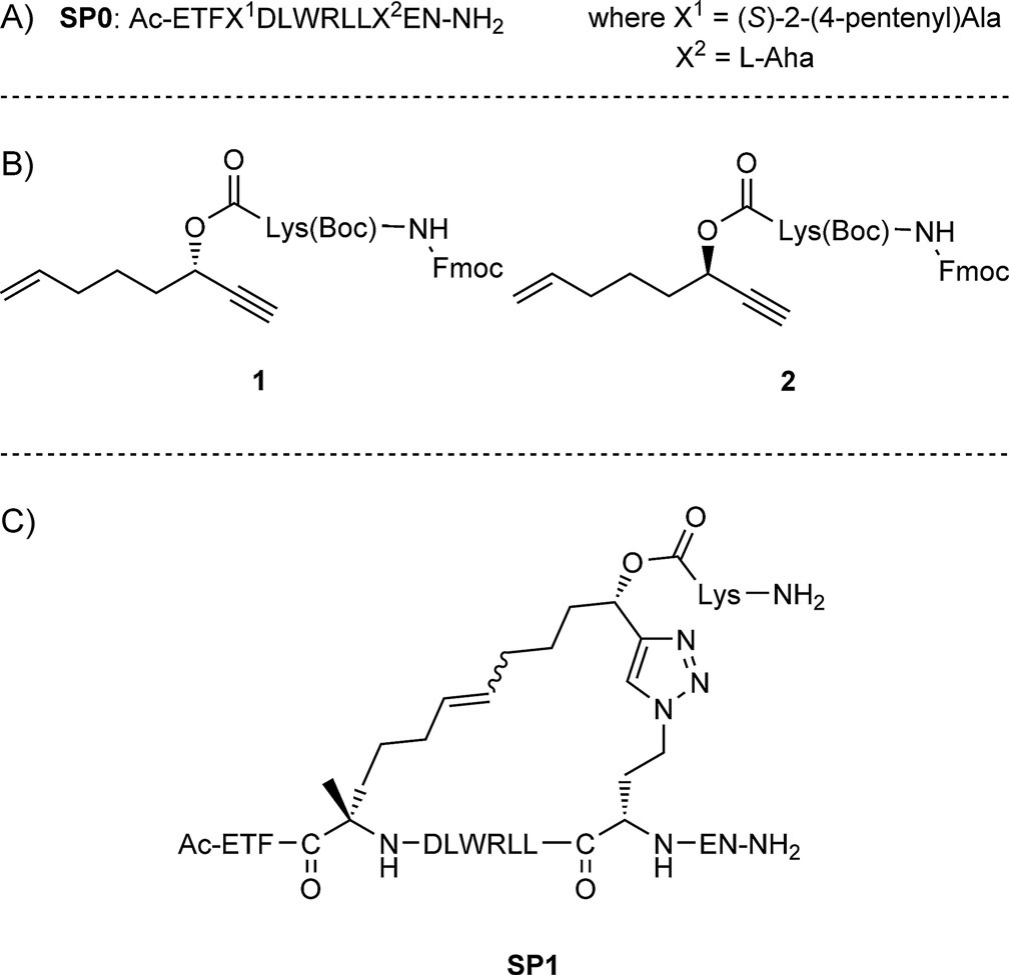
A) **SP0** (based on residues 17–29 of the p53 sequence). B) Linkers **1** and **2**. C) Structure of **SP1** after global deprotection. l‐Aha=azido‐l‐homoalanine.

These staples contain a reactive handle from which to repeatedly grow the staple directionally depending on the stereochemistry of the linker's chiral centre. This allows the rapid synthesis of a diverse library of stapled peptides starting from a single linear peptide and two linkers (Figure 1 C). The enantiopure and diverse libraries generated can then be used directly to study the effect of conformational differences in the staple chain on the biophysical properties and cellular activity of stapled peptides.

To demonstrate the applicability of this stapling strategy, we developed stapled peptides targeting the p53–MDM2 interaction. Inhibition of this PPI blocks the ubiquitylation of p53 by MDM2 and therefore its subsequent degradation, thus restoring p53 activity in cancer cells.^16^ The development of highaffinity stapled peptides has proven a successful approach for targeting this PPI, through both one‐ and two‐component strategies and with a variety of stapling chemistries.13d, 17 To develop the *i*,*i*+7 strategy, we opted to use a comparable sequence to that used by Spring and co‐workers in their two‐component, double‐click stapling methodology.^11^ Whereas they replaced Ser20 and Pro27 residues in the wild‐type p53_17–29_ peptide with azido amino acids, in our sequence **SP0** we replaced Ser20 with an olefin amino acid and Pro27 with an azido amino acid (Scheme 1 A).

As this stapling strategy employs two orthogonal reactions, the order in which the stapling reactions are performed is important for minimising side products. We first performed CuAAC first to avoid the possibility of either homodimerisation or enyne metathesis of the linker upon exposure to Grubbs' catalyst.^18^ Additionally, performing RCM second could preferentially favour the formation of a single *E* or *Z* olefin isomer. Preliminary investigations on a different α‐helical peptide sequence had identified that an eight‐atom linker would maximise conversion to the stapled peptide. Similarly, it was found that stapling tolerated substitution at the propargylic position on the linker. Using these guidelines, linkers **1** and **2** were synthesised in high yield and enantiopurity from simple building blocks to be used in our final stapled peptides (Scheme S2).

CuAAC and RCM conditions were then optimised for incorporating linkers **1** and **2** onto **SP0**. Agitating the protected and resin‐immobilised **SP0** with 2.5 equiv of linker, 3 equiv of CuSO_4_⋅5 H_2_O and 3.1 equiv of sodium ascorbate overnight in DMF/H_2_O (9:1) resulted in near quantitative conversion to the triazole product, hereafter referred to as unstapled **SP1** (Figure S4 B in the Supporting Information). Initial efforts to perform RCM on unstapled **SP1** by using Grubbs' second‐generation catalyst at room temperature resulted in little to no conversion to the fully stapled **SP1**. Heating to 50 °C yielded detectable conversion, but with the production of multiple side products. Grubbs' first‐generation catalyst resulted in high conversion after a single round of catalyst loading. Subjecting unstapled **SP1** to two rounds of Grubbs' first‐generation catalyst (6 mm, 3 equiv) in 1,2‐dichloroethene (1,2‐DCE) at room temperature generated **SP1** with no starting material observed (Figure S4 C). Using these optimised conditions, we synthesised stapled peptides **SP1** and **SP2** in two steps with minimal side product formation and excellent conversion.

Following RCM stapling, two staple products were detected in a 3:2 ratio. We suspected them to be the *E*/*Z* olefin isomers formed during RCM, as their masses were identical as determined by mass spectrometry (Table S3). Although most stapling studies using RCM result in the production of a major isomer in >90 % abundance, strategies using longer staples (i.e., *i*,*i*+7) have been shown to yield more equal ratios of *E*/*Z* isomers.15a The two isomers were separated during peptide purification and designated A or B (e.g., **SP1 (A)**) based on retention time, with A being the major isomer and the first to elute (Figure S4 C).

We incorporated additional lysine residues into the staple chain, as the addition of positively charged residues is an often‐used strategy for optimising the cell permeability of stapled peptides.^19^ FITC was likewise incorporated, as it can be used for biophysical studies. Starting from base stapled peptides **SP1** and **SP2**, we used standard SPPS conditions to attach either an additional lysine, or β‐alanine and FITC, to the pendant lysine attached to the linker's chiral centre (Scheme 2). This expansion was performed while the staple was still on‐resin and its residues protected, thereby minimising possible side reactions. Using this strategy, we generated staples **SP1‐K**, **SP2‐K**, **SP1‐βA‐F**, and **SP2‐βA‐F** with excellent conversion, no major side‐product generation and retention of A/B isomer ratios (Figure S5).

**Scheme 2:**
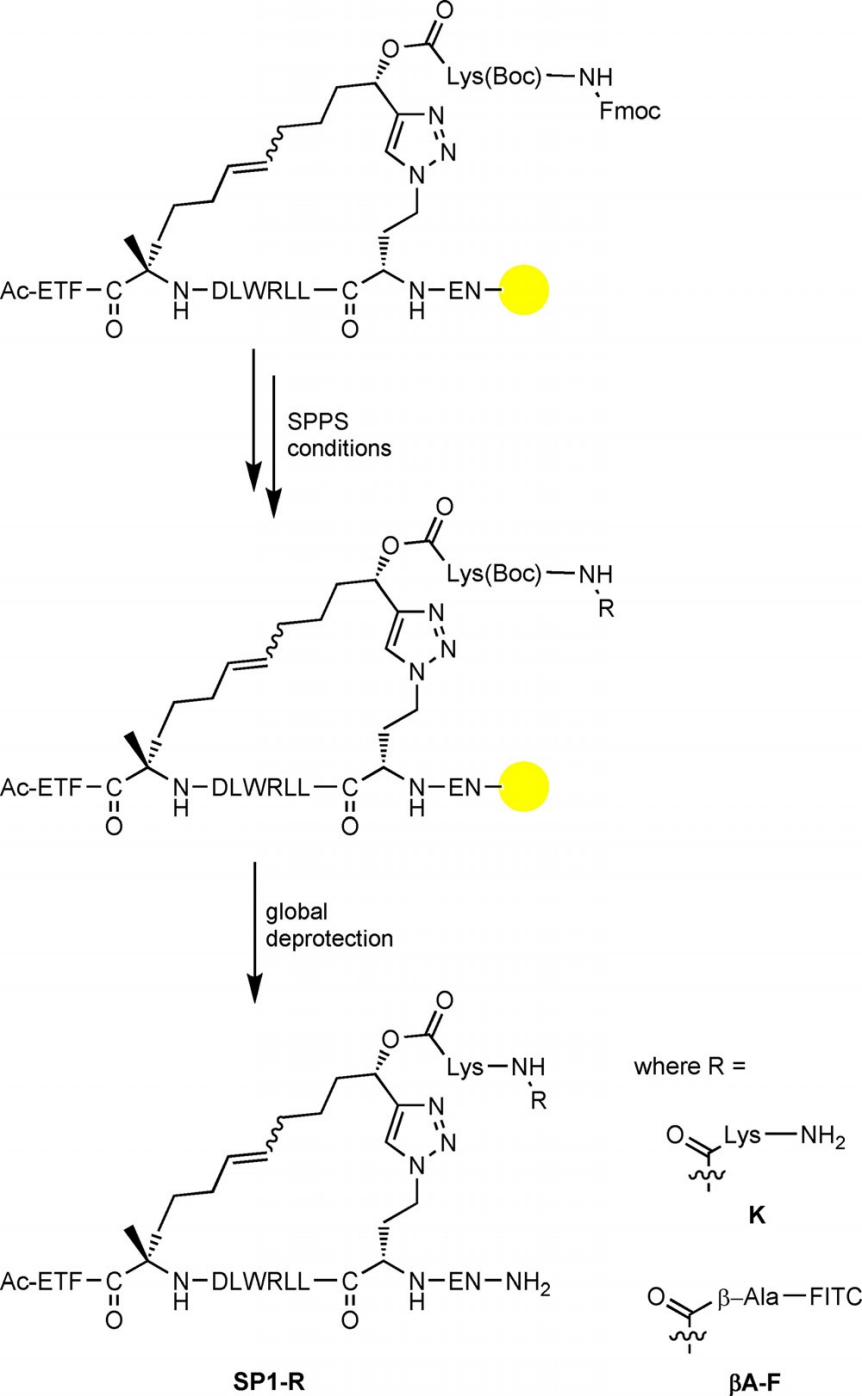
Expansion of **SP1** to **SP1‐K** and **SP1‐βA‐F** under SPPS conditions. FITC=fluorescein isothiocyanate, Lys=lysine. The yellow ball represents the MBHA resin support.

**SP0** and stapled peptides **SP1**, **SP2**, **SP1‐K**, **SP2‐K**, **SP1‐βA‐F** and **SP2‐βA‐F** were cleaved from resin, and their A and B isomers were separated and purified to >95 % purity. Each purified peptide was dissolved in phosphate buffer (PBS; 50 mm)/acetonitrile (95:5, pH 7.4) and its circular dichroism spectrum was recorded.

As expected, substituting the helicity‐disrupting Pro27 residue gave **SP0** higher helicity (52 %) than that reported for the wild‐type peptide (10 %).^11^ Comparing the A/B isomers for all stapled examples with the unstapled peptide shows a mild to moderate increase in helicity upon stapling for the A isomer and a decrease in helicity for the B isomer (Figure 2). This suggested that one of the *E*/*Z* isomers might constrict the peptide in an α‐helical conformation, whereas the other isomer disrupts the intrinsic helicity of the peptide. The A isomers of peptides stapled with linker **1**, with the exception of **SP1‐βA‐F (A)**, display negligible increases in helicity compared to **SP0**, whereas the A isomers of peptides stapled with linker **2** show substantial increases in helicity (Table 1). This pattern of higher helicity for peptides incorporating linker **2** suggests that the stereochemistry of the chiral centre in the linker used might be a factor in stabilising helicity post‐stapling. Expansion of the staple in **SP1‐K**, **SP2‐K**, **SP1‐βA‐F** and **SP2‐βA‐F** did not seem to affect helical content compared to base‐stapled peptides **SP1** and **SP2** (Figures 2 and S6). The retention of helicity across derivatives of **SP1** and **SP2** suggests that the introduction of additional functional groups to the staple chain would not interfere with the inherent helicity of the unexpanded staple.

**Figure 2:**
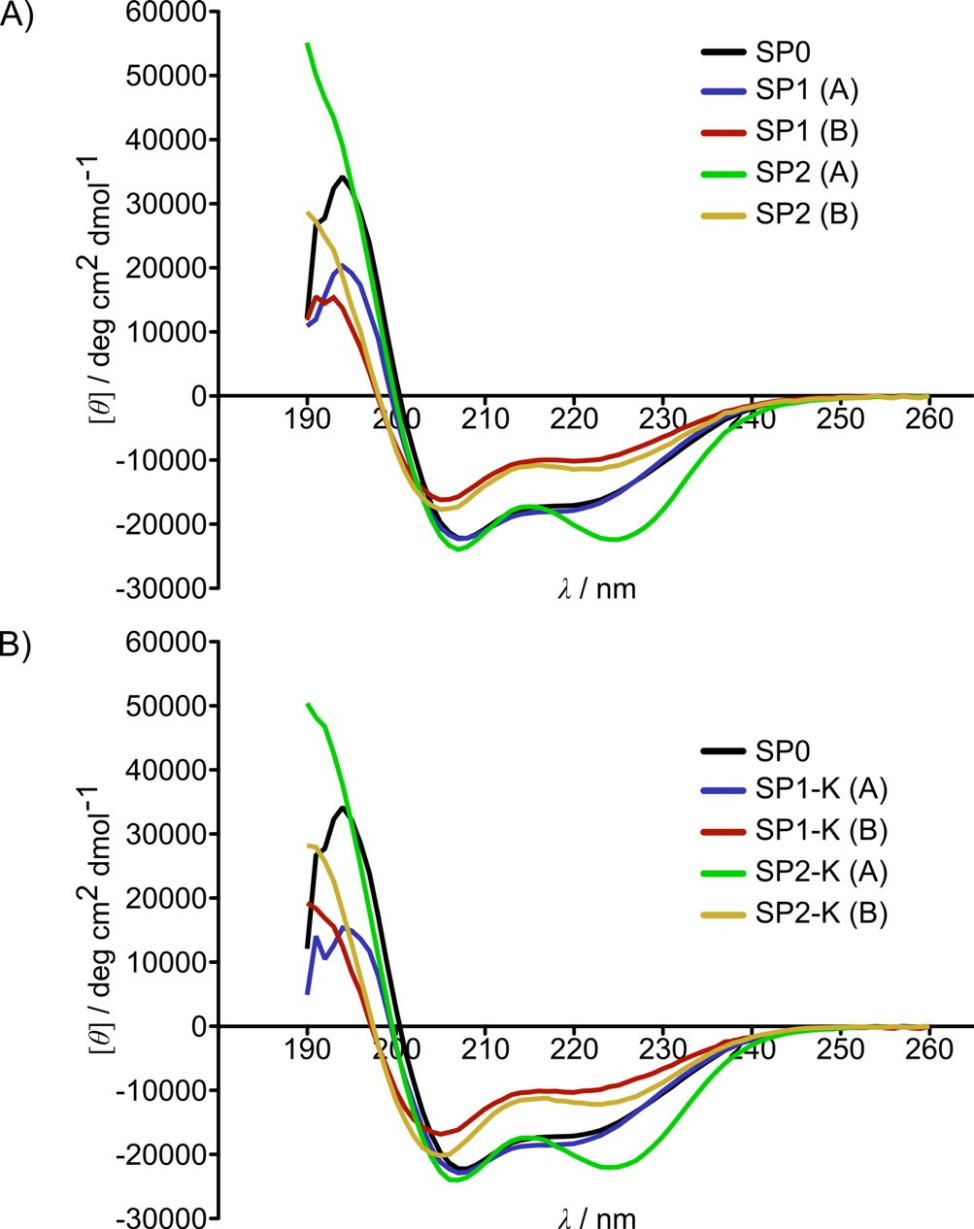
Circular dichroism spectra of peptides **SP0** and A) **SP1** and **SP2** and B) **SP1‐K** and **SP2‐K**.

**Table 1:** Binding affinities and helicity for peptides determined by competitive fluorescence polarisation and circular dichroism, respectively. Errors shown are the standard deviation of two triplicate experiments.

Peptide^[a]^	FP *K*_d_ [nm]	Helicity [%]	Peptide^[a]^	FP *K*_d_ [nm]	Helicity [%]
**SP0**	8.6±1.7	52			
**SP1 (A)**	4.5±1.3	53	**SP1‐K (A)**	5.5±1.2	55
**SP1 (B)**	12.0±1.6	31	**SP1‐K (B)**	12.0±1.7	31
**SP2 (A)**	3.8±1.0	67	**SP2‐K (A)**	11.1±2.4	65
**SP2 (B)**	30.2±6.6	35	**SP2‐K (B)**	14.7±2.0	36
			nutlin‐**3 a**	110±26.8	n.a.

[a] CD and direct FP data for **SP1‐βA‐F (A/B)** and **SP2‐βA‐F (B)** are provided in the Supporting Information. n.a.: not applicable.

Peptide stapling improved the proteolytic stability of the peptide. Peptide **SP0** displayed poor in vitro stability, with only 17 % of the peptide remaining intact after 6 h of incubation with α‐chymotrypsin (Figure 3). **SP1 (A)** showed significantly improved stability with 82 % of the peptide left intact after 6 h.

**Figure 3:**
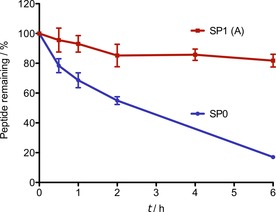
Serum protease stability of peptides **SP0** and **SP1 (A)** incubated with α‐chymotrypsin. Error bars indicate standard error of the mean based on two replicate experiments.

Both stapled and unstapled peptides showed high affinity binding for MDM2 in vitro, as determined by a competitive fluorescence polarisation assay benchmarked with nutlin‐3a, a potent inhibitor of MDM2 (Table 1).13d Their binding affinities also compare favourably to those previously reported for wild‐type p53_17–29_ peptide, which has a reported *K*_d_ of 821 nm,11a and other p53‐derived stapled peptides.^11^, 17a Stapling modestly improved the binding affinity compared with unstapled peptide **SP0** in some cases: binding affinity increased for all isomer A stapled peptides (with the exception of **SP2‐K**), whereas isomer B stapled peptides showed a decrease in binding affinity compared with **SP0**. These results correlated well with the relative helicities of isomers A and B, whereby an increase in helicity upon stapling results in an increase in binding affinity. This result suggests that the *E*/*Z* configuration of the double bond on the staple chain plays an important role in both imparting greater helicity to the peptide and improving MDM2 binding affinity. In contrast, the increased helicity of peptides stapled with linker **2** resulted in only negligible changes in binding affinity relative to those stapled with linker **1**. Expanding the staple chain by adding an extra lysine likewise did not have a significant effect on binding affinity, possibly because the side chain is not sufficiently long to reach the protein surface.

In summary, we have developed the first two‐component, *i,i*+7 stapling methodology that enables both the incorporation of linkers with a chiral centre and post‐stapling modification of the staple itself by using standard SPPS chemistry. Although a major/minor mixture of *E*/*Z* isomers was generated following RCM, the two were readily separable, with the major isomer displaying the better helicity and in vitro target–protein binding. The major stapled peptide isomers (A) exhibit increased helicity, proteolytic stability and in vitro binding to MDM2 compared with the unstapled peptide. Additionally, moderate increases in helicity after stapling correlated well with peptides derived from linker **2** in most cases, thus suggesting that linker stereochemistry might play a role in helical stability. Expanding the staple did not significantly disrupt helicity or protein binding, thus suggesting that post‐stapling modification might provide the opportunity to further improve binding to the target protein with retention of other preferential properties. We foresee this stapling methodology being applied to create diverse libraries of stapled peptides with various functionalities starting from a single stapled peptide. These stereopure libraries can then be used to study the effects of staple‐chain stereochemistry and functionality on a stapled peptide's biophysical and biochemical properties.

## Experimental Section

**Synthesis**: Linkers **1** and **2** were synthesised from readily available starting materials. Amino acid Fmoc‐l‐azidohomoalanine was synthesised as previously described.^20^ Fmoc‐(*S*)‐2‐(4‐pentenyl)alanine was purchased from Sigma–Aldrich. For schemes and synthetic procedures see the Supporting Information.

Manual peptide synthesis was performed on Novabiochem Rink Amide MBHA resin (1 mmol, 0.37 mmol g^−1^ loading). Amino acid couplings were carried out by adding a solution of *N*‐[(1*H*‐benzotriazole‐1‐yl)(dimethylamino)methylene]‐*N*‐methylmethanaminium hexafluorophosphate *N*‐oxide (HBTU; 5 equiv, 0.38 m) in DMF to a solution of Fmoc‐protected amino acid (5 equiv, 0.4 m) in DMF. After 5 min, the pre‐activated mixture was added to the resin and agitated for 1–3 h. The side‐chain‐protecting groups used were *t*Bu for aspartic acid, glutamic acid and threonine, Boc for lysine and tryptophan, Pbf for arginine and Trt for asparagine. Completion of peptide coupling was monitored by the chloranil test, in which 2 % acetaldehyde in DMF (2–4 drops) and 2 % chloranil in DMF (2–4 drops) were added to a small amount of resin and shaken for 5–10 min at RT. No change in the colour of the resin indicated complete coupling, whereas green indicated incomplete coupling. Fmoc deprotection was carried out with 25 % piperidine in DMF (2×10 min). *N*‐Acetyl capping of the completed peptide was carried out by agitating the resin in a solution of acetic anhydride/*N*,*N*‐diisopropylethylamine (DIPEA)/DMF (1:0.25:10, *v*/*v*/*v*) for 1 h at RT. Cleavage from the resin and global deprotection was achieved with a mixture of 5 % H_2_O, 2 % triisopropylsilane and 5 % phenol in trifluoroacetic acid (TFA; 0.1 mL/10 mg of resin) for 2 h at RT. The solvent was removed under a stream of argon, and the residue was triturated with ice‐cold diethyl ether (2×1.5 mL) before HPLC purification.

**Peptide stapling**: on‐resin **SP0** (1 equiv) followed by CuSO_4_⋅5 H_2_O (3 equiv) in DMF (4.5 mL) was added to a plastic syringe fitted with a stopper. Linker (2.5 equiv) was then added to the reaction mixture, followed by sodium ascorbate (3.1 equiv) in H_2_O (0.5 mL), whereupon the solution turned yellow. The vessel was closed with a syringe cap and agitated overnight at RT. The mixture was then filtered, and the resin washed with CH_2_Cl_2_ (3×5 mL). Resin was then swelled in 1,2‐DCE (2 mL) for 10 min in a plastic frit. The solvents were drained, and the resin was washed with 1,2‐DCE (2×5 mL). A solution of Grubbs' first‐generation catalyst (3 equiv, 6 mm) in anhydrous 1,2‐DCE was added, and argon was slowly bubbled through the mixture for 2 h at RT. Care was taken to maintain a constant volume of 1,2‐DCE. The solvents were drained, and the resin was washed with 1,2‐DCE (2×5 mL) to remove any residual reaction solution. A second amount of catalyst was then loaded as described above for an additional 2 h. The solvents were drained, and the resin was washed with 1,2‐DCE (3×5 mL) and CH_2_Cl_2_ (3×5 mL), then shrunk by washing with MeOH (3×5 mL).

**Peptide staple expansion: SP1** or **SP2** was subjected to standard SPPS for the addition of lysine or β‐alanine. Coupling with FITC was achieved by stirring on‐resin peptide (1 equiv) with a solution of FITC (1.1 equiv) in pyridine/DMF/CH_2_Cl_2_ (12:7:5, *v*/*v*/*v*) overnight at RT. All stapled peptides with a terminal Fmoc‐protected lysine on the staple were Fmoc‐deprotected under the conditions described above prior to cleavage from resin.

**HPLC analysis and purification**: Analytical reversed‐phase HPLC was performed on an Agilent 1100 Series HPLC System using a silica μBondapak C_18_ Column (125 Å, 10 μm, 150×3.9 mm) and eluting with a linear‐gradient system (solvent A: 0.1 % (*v*/*v*) TFA in water→solvent B: 0.1 % (*v*/*v*) TFA in acetonitrile) over 25 min at a flow rate of 1 mL min^−1^. Semi‐preparative reversed‐phase HPLC was performed on the same system using a silica YMC‐Pack Pro C_18_ column (120 Å, 10 μm, 250×10 mm) and eluting with a linear‐gradient system (A→B) over 25 min at a flow rate of 5 mL min^−1^. HPLC was monitored by the UV absorbance at 220 nm.

**Circular dichroism**: CD spectra were obtained on a Chirascan CD spectrometer at 20 °C (1 mm path length, 260–190 nm, bandwidth 1.0 nm, response time 0.5 s, step resolution 1.0 nm) Each spectrum is the average of three scans. Peptides were dissolved in PBS (50 mm)/acetonitrile (95:5, pH 7.4) at 20 °C. The helicity was calculated as previously reported19b by comparing mean residue ellipticity at 222 nm (MRE_222_) to the theoretical maximum as calculated from the formula [Eq. (1), *n*=number of amino acid residues] described by Forood et al.^21^

(1) $$\mathrm{MRE}{}_{222}=-40\;000\times \left(1-2.5/n\right)$$

Spectra were obtained at several concentrations (30–215 mm). No significant change in the shape of the spectrum was observed for the different dilutions. Accurate peptide concentrations were determined by amino acid analysis at the Peptide Nucleic Acid Chemistry Facility (Department of Biochemistry, University of Cambridge).

**Serum stability assay**: Each peptide was dissolved in PBS (300 μL) from a DMSO stock to a final concentration of 0.3 mm. This PBS contained α‐chymotrypsin (0.5 μg mL^−1^) and caffeine as an internal standard. Peptides were incubated at RT, and at each time point, an aliquot (30 μL) of the mixture was removed, snap frozen, thawed and centrifuged, and the supernatant was analysed by HPLC. Peptide degradation was monitored by comparing the integration of the peptide peak against the internal standard at 220 nm. Experiments were performed in duplicate.

**Preparation of recombinant MDM2**: MDM2 (residues 2–125) was expressed and purified according to described procedures.^22^

**Fluorescence polarisation assays**: Fluorescence polarisation was performed in 384‐well microplates (Corning) with a CLARIOstar microplate reader (BMG labtech, Germany). An assay buffer containing PBS, Tween 20 (0.1 % *v*/*v*) and dithiothreitol (DTT; 2 mm) was used. Accurate peptide concentrations were determined by amino acid analysis at the Peptide Nucleic Acid Chemistry Facility (Department of Biochemistry, University of Cambridge). Direct and competitive FP procedures, binding affinity calculations and binding curves are provided in the Supporting Information.

## Conflict of interest

*The authors declare no conflict of interest*.

## Supporting information

As a service to our authors and readers, this journal provides supporting information supplied by the authors. Such materials are peer reviewed and may be re‐organized for online delivery, but are not copy‐edited or typeset. Technical support issues arising from supporting information (other than missing files) should be addressed to the authors.

Supplementary
