# Supplementary material for: A New Methodology for Incorporating Chiral Linkers into Stapled Peptides
Source: Chembiochem. 2017 May 18;18(12):1066–71. doi: 10.1002/cbic.201700075 (PMC5488175; doi:10.1002/cbic.201700075)
Supplement: Supplementary file 1 — Supplementary [file CBIC-18-1066-s001.pdf]

CHEMBIOCHEM

Supporting Information

A New Methodology for Incorporating Chiral Linkers into Stapled Peptides

Juan C. Serrano,[a] James Sipthorp,*[a] Wenshu Xu,[b] Laura S. Itzhaki,[b] and Steven V. Ley[a]

cbic_201700075_sm_miscellaneous_information.pdf

Supporting Information

1. General Experimental Details
2. Synthetic Procedures
3. Fluorescence Polarisation Assays
4. NMR Spectra
5. Peptide Characterisation
6. References

1. General Experimental Details

All reactions were performed using oven-dried glassware (140 °C) under an atmosphere of dry argon unless using aqueous reagents or otherwise stated. All solvents used in reactions were dried and distilled using standard procedures. Dimethylformamide (DMF) for peptide synthesis was purchased from Sigma-Aldrich. Imidazole-1-sulfonyl azide hydrochloride was synthesised according to literature procedure.[1] All other reagents were used as supplied or purified using standard procedures as necessary.

Flash column chromatography (FCC) was performed using Breckland Scientific silica gel 60, particle size 40–63 nm under air pressure. All solvents used for chromatographic purification were distilled prior to use. Analytical thin layer chromatography (TLC) was performed using silica gel 60 F254 pre-coated glass backed plates and visualised by ultraviolet radiation (254 nm) and/or potassium permanganate as appropriate.

Quantities are reported to 3 significant figures and are rounded accordingly. Isolated yields are reported to 0 decimal places (DP) and "quant." signifies a yield of 99.5% or higher.

^{1}H NMR spectra were recorded on a Bruker DRX-600 (600 MHz). Chemical shifts are reported in ppm with the resonance resulting from incomplete deuteration of the solvent as the internal standard ($CDCl_3$: 7.26 ppm; DMSO-d_6: 2.50 ppm, quint; D_2O: 4.79 ppm). ^{13}C NMR spectra were recorded on a Bruker DRX-600 (150 MHz) spectrometer with complete proton decoupling. Chemical shifts are reported in ppm with the solvent resonance as the internal standard ($^{13}CDCl_3$: 77.16 ppm, t; ^{13}DMSO-d_6: 39.52 ppm, sept). All ^{1}H and ^{13}C spectra were recorded at 298 K unless otherwise specified. Data are reported as follows: chemical shift δ/ppm (multiplicity (s = singlet, d = doublet, t = triplet, q = quartet, quint = quintet, sext = sextet, sept = septet, br = broad, m = multiplet or combinations thereof. ^{13}C signals are singlets unless otherwise stated), coupling constants *J* Hz, integration (^{1}H only), assignment). Spectra are assigned as fully as possible, using ^{1}H-COSY, ^{13}C-DEPT, HMQC, HMBC and NOESY where appropriate to facilitate structural determination. Signals that could not be unambiguously assigned are reported with all possible assignments separated by a slash (e.g. H^1/H^2). Multiple signals arising from diastereotopic or (pseudo-)axial/equatorial positions are suffixed alphabetically (e.g. H^{1a}, H^{1b}). Signals arising from mixtures of stereoisomers are suffixed with maj (major isomer) or min (minor isomer). Overlapping signals that could not be resolved are reported with their assignments denoted in list format (e.g. H^1, H^2, H^3). ^{1}H are reported to 2 DP and ^{13}C NMR signals to 1 DP unless necessary to distinguish similar signals.

High resolution mass spectrometry (HRMS) was performed on a Waters LCT Premier spectrometer using electrospray ionization, time-of-flight analysis and Masslynx version 4.0 software. Matrix assisted laser desorption/ionization time of flight mass spectrometry (MALDI-TOF) was performed on an Applied Biosystems 4700 MALDI-TOF. Ultra-performance liquid chromatography mass-spectrometry (UPLC-MS) was performed on a Water Xevo G2-S QTof with a Waters I-class AQUITY UPLC. Mass values are reported up to 4 DP and are within the error limits of ±5 ppm.

Infrared spectra were recorded neat as thin films on a Perkin-Elmer Spectrum One FTIR spectrometer and only selected peaks are reported (s = strong, m = medium, w = weak, b = broad, sh = sharp).

Melting points were collected using a Stanford Research Systems Optimelt automated melting point system using a gradient of 1 °C per min.

Optical rotation was measured on a PerkinElmer Model 343 Polarimeter. $[\alpha]_D$ values are reported in 10^{-1} deg cm^2 g^{-1} at 589 nm, with concentration (c) given in g/100 mL.

Chiral normal-phase HPLC was run on an Agilent 1100 Series HPLC System using a silica Chiralpak AD-H column (5 µm, 250 × 4.6 mm) eluting with a linear gradient system (solvent A: hexanes, solvent B: isopropyl alcohol). HPLC was monitored by UV absorbance at 210 nm or 230 nm. Retention times (t_R) are reported to the nearest 0.001 min.

Analytical reverse-phase HPLC was run on an Agilent 1100 Series HPLC System using a silica µBondapak C18 Column (125 Å, 10 µm, 150 × 3.9 mm) eluting with a linear gradient system (solvent A: 0.1% (v/v) TFA in water, solvent B: 0.1% (v/v) TFA in acetonitrile) over 25 min at a flow rate of 1 mL/min. Semi-preparative reverse-phase HPLC was run on the same system using a silica YMC-Pack Pro C18 column (120 Å, 10 µm, 250 × 10 mm) eluting with a linear gradient system (solvent A: 0.1% (v/v) TFA in water, solvent B: 0.1% (v/v) TFA in acetonitrile) over 25 min at a flow rate of 5 mL/min. HPLC was monitored by UV absorbance at 220 nm. Retention times (t_R) are reported to the nearest 0.001 min.

Peptide concentrations were determined by amino acid analysis at the Peptide Nucleic Acid Chemistry Facility at the Department of Biochemistry, University of Cambridge.

2. Synthetic Procedures

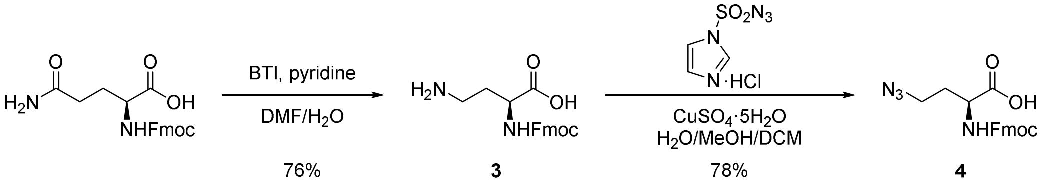

Scheme S1. Synthesis of amino acid Fmoc-L-Aha-OH (**4**).

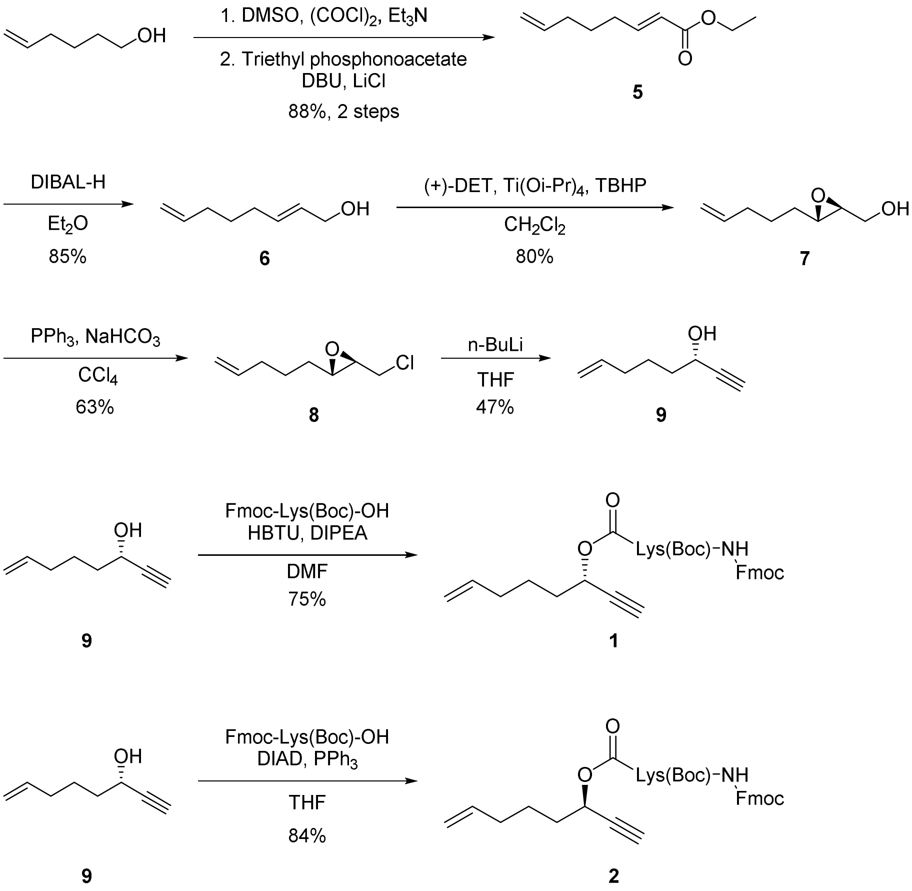

Scheme S2. Synthesis of linkers **1** and **2**

(S)-2-((((9H-fluoren-9-yl)methoxy)carbonyl)amino)-4-aminobutanoic acid (3)

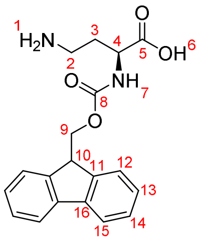

[Bis(trifluoroacetoxy)iodo]benzene (9.34 g, 22 mmol) was dissolved in a 2:1 mixture of DMF:H_2O (96 mL). Fmoc-Gln-OH (5.00 g, 14 mmol) was then added and the mixture stirred for 15 min. Pyridine (2.70 mL, 33 mmol) was then added and the reaction mixture stirred for 14 h at rt. DMF was then distilled from the mixture leaving behind an orange liquid which was dissolved in H_2O (75 mL) and acidified with 12 M HCl (1.5 mL). The solution was washed with Et_2O (4 × 50 mL) and the aqueous layer adjusted to pH 6 with 10% aq. NaOH, resulting in a white precipitate. The precipitate was filtered, washed with H_2O (3 × 30 mL), ice-cold EtOH (15 mL), Et_2O (10 × 10 mL) and dried *in vacuo* to afford **3** (3.50 g, 76%) as a white solid and used immediately in the next step.

^{1}H NMR (600 MHz, DMSO-d_6) δ 7.89 (d, J = 7.5 Hz, 2H, H^{15}), 7.68 (d, J = 7.2 Hz, 2H, H^{12}), 7.42 (t, J = 7.5 Hz, 2H, H^{14}), 7.33 (t, J = 7.6 Hz, 2H, H^{13}), 6.64 (d, J = 4.0 Hz, 1H, H^7), 4.32–4.17 (m, 3H, H^9, H^{10}), 3.66–3.57 (m, 1H, H^4), 2.99–2.80 (m, 2H, H^2), 1.93–1.83 (m, 1H, H^{3a}), 1.74–1.65 (m, 1H, H^{3b}); HRMS (ESI+) calculated for $C_{19}H_{21}N_2O_4$ $[M+H]^+$ 341.1501, found 341.1503. Characterisation data is in accordance with that previously reported.[2]

(S)-2-((((9H-fluoren-9-yl)methoxy)carbonyl)amino)-4-azidobutanoic acid (4)

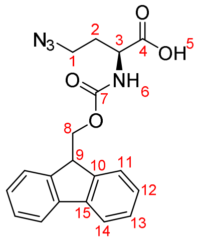

To a solution of **3** (2.00 g, 5.88 mmol) in a biphasic mixture of H_2O (30 mL), MeOH (60 mL) and CH_2Cl_2 (50 mL) was added $CuSO_4{\cdot}5H_2O$ (14.7 mg, 1 mol%) and imidazole-1-sulfonyl azide hydrochloride (3.81 g, 18.2 mmol). The reaction mixture was adjusted to pH 9 with sat. K_2CO_3 and stirred for 18 h at rt. The reaction was then diluted with CH_2Cl_2 (60 mL) and the aqueous phase separated. The organic layer was then extracted with sat $NaHCO_3$ (2 × 75 mL). The aqueous extracts were combined and washed with Et_2O (2 × 75 mL), then acidified to pH 2 with conc. HCl. The solution was then extracted with Et_2O (3 × 75 mL), dried over $MgSO_4$ and concentrated *in vacuo* to afford **4** (1.67 g, 78%) as a white solid.

^{1}H NMR (600 MHz, $CDCl_3$) δ 7.77 (d, J = 7.5 Hz, 2H, H^{14}), 7.59 (d, J = 6.9 Hz, 2H, H^{11}), 7.41 (t, J = 7.4 Hz, 2H, H^{13}), 7.32 (t, J = 7.4 Hz, 2H, H^{12}), 5.41 (d, J = 7.4 Hz, 1H, H^6), 4.65–4.38 (m, 3H, H^3, H^8), 4.23 (t, J = 6.6 Hz, 1H, H^9), 3.47–3.38 (m, 2H, H^1), 2.26–2.13 (m, 1H, H^{2a}), 2.06–1.94 (m, 1H, H^{2b}); ^{13}C NMR (150 MHz, $CDCl_3$) δ 176.1 (C^4), 156.3 (C^7), 143.7 (C^{10}), 141.5 (C^{15}), 127.9 (C^{14}), 127.2 (C^{13}), 125.1 (C^{12}), 120.2 (C^{11}), 67.3 (C^8), 51.8 (C^3), 47.8 (C^1), 47.3 (C^9), 31.4 (C^2); $[\alpha]_D^{24}$ = −14.6 (c = 0.7, DMSO), Lit[2] = −14.4 (c = 1, MeOH); FTIR (neat, υ_{max}, cm^{-1}) 3332 (m), 3200 (w), 2750 (w), 2105 (w), 1693 (s), 1553 (w); HRMS (ESI+) calculated for $C_{19}H_{19}N_4O_4$ $[M+H]^+$ 367.1420, found 367.1420. Characterisation data is in accordance with that previously reported.[2]

(E)-ethyl octa-2,7-dienoate (5)

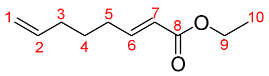

DMSO (8.88 mL, 125 mmol) was added to a stirred solution of oxalyl chloride (5.90 mL, 70 mmol) in CH_2Cl_2 (250 mL) at −78 °C under argon atmosphere. The reaction mixture was stirred for 18 min then a solution of 5-hexen-1-ol (6.00 mL, 50 mmol) in CH_2Cl_2 (65 mL) was added slowly to the mixture. The mixture was stirred for 18 min, then triethylamine (34.9 mL, 250 mmol) was added. After stirring for 0.5 h at −78 °C, the reaction mixture was allowed to warm up to room temperature and stirred for an additional 3 h. A solution of lithium chloride (3.82 g, 90 mmol), 1,8-diazabicyclo[5.4.0]undec-7-ene (13.5 mL, 90 mmol) and triethyl phosphonoacetate (17.9 mL, 90 mmol) in MeCN (175 mL) was prepared separately and stirred for 1 h. The reaction mixture was then concentrated *in vacuo* and the prepared solution was added to the flask and the mixture was stirred overnight at rt. The reaction was then quenched with a solution of sat. NH_4Cl (45 mL) and concentrated *in vacuo*, leaving an orange residue. The residue was then extracted with Et_2O (2 × 60 mL) and the organic layers were combined. The solution was then washed with water (2 × 50 mL), brine (2 × 50 mL), dried over $MgSO_4$ and

concentrated *in vacuo* to give an orange liquid. Further purification by column chromatography (hexanes:EtOAc, 9:1) gave **5** (7.28 g, 88%) as a pale yellow liquid.

^{1}H NMR (600 MHz, $CDCl_3$) δ 6.96 (dt, *J* = 15.6, 7.0 Hz, 1H, H^6), 5.84–5.74 (m, 2H, H^2, H^7), 5.02 (dd, *J* = 17.1, 1.7 Hz, 1H, H^1-*trans*), 4.98 (dd, *J* = 10.2, 1.7 Hz, 1H, H^1-*cis*), 4.18 (q, *J* = 7.1 Hz, 2H, H^9), 2.21 (td, *J* = 8.0, 6.9 Hz, 2H, H^5), 2.08 (td, *J* = 7.4, 7.2 Hz, 2H, H^3), 1.56 (p, *J* = 7.5 Hz, 2H, H^4), 1.29 (t, *J* = 7.1 Hz, 3H, H^{10}); ^{13}C NMR (150 MHz, $CDCl_3$) δ 166.7 (C^8), 148.9 (C^6), 138.0 (C^2), 121.5 (C^7), 115.1 (C^1), 60.2 (C^9), 33.1 (C^3), 31.5 (C^5), 27.2 (C^4), 14.3 (C^{10}); FTIR (neat, υ_{max}, cm^{-1}) 2984 (w), 2934 (w), 2857 (w), 1718 (sh), 1654 (m), 1443 (w), 1368 (m), 1265 (s), 1196 (s), 1173 (s), 1039 (s), 976 (s), 911 (s), 869 (w), 710 (w); R_f 0.69 (7:3 hexanes:EtOAc); HRMS (ESI+) calculated for $C_{10}H_{17}O_2$ $[M+H]^+$ 169.1229, found 169.1227. Characterisation data is in accordance with that previously reported.[3]

(*E*)-octa-2,7-dien-1-ol (6)

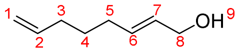

5 (6.90 g, 41.0 mmol) was dissolved in Et_2O (220 mL) under argon atmosphere, then cooled to −78 °C. DIBAL-H (16.4 mL, 91.5 mmol) was then added dropwise over 15 min, then the reaction mixture was stirred for 3 h. The reaction was then slowly quenched with a sat. solution of potassium sodium tartrate (300 mL) and stirred vigorously for 2 h, whereupon two clear, separate phases were observed. The aqueous phase was separated and the organic phase filtered through celite. The organic filtrate was then concentrated *in vacuo* to 50 mL, washed with water (2 × 50 mL), brine (2 × 50 mL), dried over $MgSO_4$ and concentrated *in vacuo* to afford a pale yellow liquid. Further purification by column chromatography (Pet. ether:Et_2O, 9:1) gave **6** (4.41 g, 85%) as a colourless liquid.

^{1}H NMR (600 MHz, $CDCl_3$) δ 5.80 (ddt, *J* = 16.9, 10.2, 6.7 Hz, 1H, H^2), 5.73–5.61 (m, 2H, H^6, H^7), 5.01 (dd, *J* = 17.1, 1.6 Hz, 1H, H^1-*trans*), 4.96 (dd, *J* = 10.2, 1.6 Hz, 1H, H^1-*cis*), 4.09 (d, *J* = 4.6 Hz, 2H, H^8), 2.10–2.03 (m, 4H, H^3, H^5), 1.49 (p, *J* = 7.5 Hz, 2H, H^4), 1.25 (br s, 1H, H^9); ^{13}C NMR (150 MHz, $CDCl_3$) δ 138.6 (C^2), 133.0 (C^7), 129.2 (C^6), 114.6 (C^1), 63.7 (C^8), 33.2 (C^5), 31.6 (C^3), 28.3 (C^4); FTIR (neat, υ_{max}, cm^{-1}) 3305 (b), 2927 (m), 1641 (w), 1439 (w), 1089 (m), 993 (m), 968 (s), 909 (s); R_f 0.42 (1:1 hexanes:EtOAc); HRMS (ESI+) calculated for $C_8H_{14}ONa$ $[M+Na]^+$ 149.0937, found 149.0934. Characterisation data is in accordance with that previously reported.[3]

(2*S*,3*S*)-2,3-epoxy-7-octen-1-ol (7)

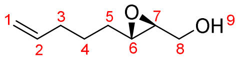

(+)-Diethyl L-tartrate (6.90 mL,39.4 mmol) was added to a solution of crushed molecular sieves (4Å MS) in CH_2Cl_2 (280 mL) under argon atmosphere at rt and then the mixture was cooled to −20 °C. Ti(OPr-*i*)$_4$ (9.90 mL, 33.3 mmol) was slowly added and the mixture was stirred for 15 min. Compound **6** (3.49 g, 27.7 mmol) was then slowly added and the reaction mixture was stirred for an additional 30 min. TBHP (11.1 mL, 61.0 mmol, 5.5 M in decane) was added dropwise over 15 min and stirred vigorously for 3 h at −20 °C. The reaction was quenched by the addition of ice-cold soln. of 15% citric acid (w/v) and 30% $FeSO_4 \cdot 7H_2O$ (w/v) (230 mL) and stirred vigorously for 30 min at 0 °C. The organic layer was separated, washed with water (2 × 100 mL), brine (2 × 100 mL), dried over $MgSO_4$, then concentrated *in vacuo* to give a pale yellow residue. The residue was then diluted with Et_2O (150 mL) and stirred with a solution of 15% NaOH (w/v) in brine (160 mL) at 0 °C for 1 h. The organic layer was separated, washed with water (2 × 100 mL), brine (2 × 100 mL), dried over $MgSO_4$, then concentrated *in vacuo* to afford a colourless liquid. Further purification by column chromatography (hexanes:EtOAc, 6:4) gave **7** (3.16 g, 80%) as a colourless liquid.

^{1}H NMR (600 MHz, $CDCl_3$) δ 5.79 (ddt, *J* = 16.9, 10.1, 6.7 Hz, 1H, H^2), 5.02 (dd, *J* = 17.1, 1.5 Hz, 1H, H^1-*trans*), 4.97 (dd, *J* = 10.2, 1.5 Hz, 1H. H^1-*cis*), 3.94–3.89 (m, 1H, H^{8a}), 3.66–3.60 (m, 1H,H^{8b}), 2.98–2.94 (m, 1H, H^7), 2.93–2.90 (m, 1H, H^6), 2.11 (dt, *J* = 6.5, 6.2 Hz, 2H, H^3), 1.71 (sb, 1H, H^9), 1.64–1.49 (m, 4H, H^4,H^5); ^{13}C NMR (150 MHz, $CDCl_3$) δ 138.2 (C^2), 115.0 (C^1), 61.6 (C^8), 58.4 (C^7), 55.7 (C^6), 33.4 (C^3), 30.9 (C^4), 25.2 (C^5); $[\alpha]_D^{27}$ = −40.4 (c = 1, $CHCl_3$), Lit[4] = −34.0 (c = 1, CH_2Cl_2); FTIR (neat, υ_{max}, cm^{-1}) 3417 (b), 3077 (w), 2977 (w), 2928 (m), 2860 (w), 1641 (m), 1458 (m), 1229 (w), 1088 (m), 1027 (m), 1039 (m), 993 (s), 909 (s), 890 (s), 765 (w); R_f 0.32 (1:1 hexanes:EtOAc); HRMS (ESI+) calculated for $C_8H_{15}O_2$ $[M+H]^+$ 143.1067, found 143.1066; The enantiomeric excess was determined to be 97% by HPLC analysis of its *p*-toluenesulfonate ester and comparison with the racemic *p*-toluenesulfonate ester. HPLC (Chiralpak AD-H column; 25 cm): 25°C, 230 nm; 0.75 mL/min; 9:1 hexanes/isopropyl alcohol; t_R (2*S*,3*S*)=16.585 min; t_R (2*R*,3*R*)=17.813 min. Characterisation data is in accordance with that previously reported.[4]

(2*R*,3*S*)-2-(chloromethyl)-3-(pent-4-en-1-yl)oxirane (8)

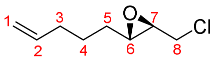

Triphenylphosphine (5.17 g, 19.7 mmol) and $NaHCO_3$ (236 mg, 2.8 mmol) were added to CCl_4 (28 mL). Compound **7** (2.00 g, 14.1 mmol) was then added and the reaction mixture was heated under reflux for 4 h. The solvents were

evaporated *in vacuo* to give a white residue which was then purified by column chromatography (hexanes:EtOAc, 95:5) which gave **8** (1.42 g, 63%) as a colourless liquid.

^{1}H NMR (600 MHz, $CDCl_3$) δ 5.79 (ddt, *J* = 16.9, 10.2, 6.7 Hz, 1H, H^2), 5.03 (dd, *J* = 17.1, 1.3 Hz, 1H, H^1-*trans*), 4.98 (dd, *J* = 10.2, 1.3 Hz, 1H, H^1-*cis*), 3.59 (dd, *J* = 11.6, 5.7 Hz, 1H, H^{8a}), 3.50 (dd, *J* = 11.6, 5.4 Hz, 1H, H^{8b}), 3.00 (td, *J* = 5.6, 1.9 Hz, 1H, H^7), 2.87 (td, *J* = 5.4, 1.9 Hz, 1H, H^6), 2.12 (dt, *J* = 6.6, 6.2 Hz, 2H, H^3), 1.63–1.49 (m, 4H, H^4, H^5); ^{13}C NMR (150 MHz, $CDCl_3$) δ 138.1 (C^2), 115.1 (C^1), 59.0 (C^7), 57.1 (C^6), 44.8 (C^8), 33.3 (C^3), 30.8 (C^4), 25.1 (C^5); $[\alpha]_D^{27}$ = −18.6 (c = 1, $CHCl_3$); FTIR (neat, υ_{max}, cm^{-1}) 3079 (w), 2977 (w), 2937 (m), 2861 (w), 1641 (sh), 1445 (m), 1264 (m), 993 (m), 912 (s), 886 (m), 730 (s); R_f 0.39 (9:1 hexanes:EtOAc); HRMS (ESI+) calculated for $C_8H_{13}OClNa$ $[M+Na]^+$ 183.0547, found 183.0541.

(*S*)-oct-7-en-1-yn-3-ol (9)

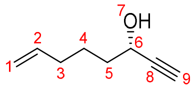

A solution of compound **8** (1.20 g, 7.47 mmol) in anhydrous THF (2.4 mL) was cooled to −40 °C. *n*-BuLi (11.3 mL, 22.4 mmol, 2 M in hexanes) was then added dropwise over 15 min and the reaction mixture was stirred for 3 h. The reaction was then quenched with NH_4Cl (6 mL) at −40 °C and allowed to stir for 30 min while warming to rt. The solvents were evaporated and the residue was diluted with Et_2O (30 mL), washed with water (2 × 30 mL), brine (2 × 30 mL), dried over $MgSO_4$, then concentrated *in vacuo* to afford a colourless liquid. Further purification by column chromatography (hexanes:EtOAc, 8:2) gave **9** (438 mg, 47%) as a colourless liquid.

^{1}H NMR (600 MHz, $CDCl_3$) δ 5.79 (ddt, *J* = 16.9, 10.2, 6.7 Hz, 1H, H^2), 5.01 (dd, *J* = 17.1, 3.3 Hz, 1H, H^1-*trans*), 4.95 (dd, *J* = 10.2, 3.3 Hz, 1H, H^1-*cis*), 4.37 (td, *J* = 6.6, 2.1 Hz, 1H, H^6), 2.45 (d, *J* = 2.1 Hz, 1H, H^9), 2.09 (dt, *J* = 7.2, 7.2 Hz, 2H, H^3), 1.76 (d, *J* = 5.5 Hz, 1H, H^7), 1.75–1.66 (m, 2H, H^5), 1.60–1.52 (m, 2H, H^4); ^{13}C NMR (150 MHz, $CDCl_3$) δ 138.3 (C^2), 114.9 (C_1), 84.8 (C^8), 73.0 (C^9), 62.2 (C^6), 37.0 (C^5), 33.2 (C^3), 24.2 (C^4); $[\alpha]_D^{28}$ = −6.1 (c = 1, $CHCl_3$); FTIR (neat, υ_{max}, cm^{-1}) 3303 (m), 3078 (w) 2932 (m), 2863 (m), 2122 (w), 1641 (m), 1030 (s), 995 (s), 911 (s); R_f 0.41 (7:3 hexanes:EtOAc); HRMS (ESI+) calculated for $C_8H_{13}O$ $[M+H]^+$ 125.0961, found 125.0960.

(*S*)-oct-7-en-1-yn-3-yl *N*^{2}-(((9*H*-fluoren-9-yl)methoxy)carbonyl)-*N*^{6}-(*tert*-butoxycarbonyl)-*L*-lysinate (1)

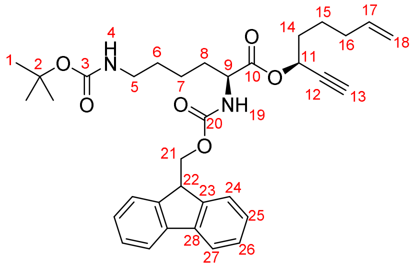

Fmoc-Lys(Boc)-OH (382 mg, 0.813 mmol) was added to a solution of **9** (100 mg, 0.805 mmol) in DMF (6.2 mL). HBTU (459 mg, 1.21 mmol) was added and the mixture was stirred for 5 min, followed by addition of DIPEA (211 µL, 1.21 mmol). The reaction mixture was stirred overnight, followed by quenching with water (6 mL). The mixture was extracted with Et_2O (30 mL), washed with LiCl (2 × 30 mL), water (2 × 30 mL), brine (2 × 30 mL), dried over $MgSO_4$ and concentrated *in vacuo* to give an off-white residue. Further purification by column chromatography (hexanes:EtOAc, 7:3) gave linker **1** (349 mg, 75%) as an off-white solid.

^{1}H NMR (600 MHz, $CDCl_3$) δ 7.76 (d, *J* = 7.6 Hz, 2H, H^{27}), 7.63–7.56 (m, 2H, H^{24}), 7.40 (t, *J* = 7.5 Hz, 2H, H^{26}), 7.32 (t, *J* = 7.5 Hz, 2H, H^{25}), 5.83–5.73 (m, 1H, H^{17}), 5.46 (d, *J* = 7.2 Hz, 1H, H^{19}), 5.39 (t, *J* = 5.8 Hz, 1H, H^{11}), 5.03 (d, *J* = 17.1 Hz, 1H, H^{18}-*trans*), 4.98 (d, *J* = 10.1 Hz, 1H, H^{18}-*cis*), 4.58 (s, 1H, H^4), 4.45–4.33 (m, 3H, H^9, H^{21}), 4.23 (t, *J* = 7.0 Hz, 1H, H^{22}), 3.16–3.03 (m, 2H, H^5), 2.51 (s, 1H, H^{13}), 2.10 (dt, *J* = 13.9, 6.9 Hz, 2H, H^{16}), 1.93–1.71 (m, 4H, H^8, H^{14}), 1.61–1.48 (m, 6H, H^6, H^7, H^{15}), 1.43 (s, 9H, H^1); ^{13}C NMR (150 MHz, $CDCl_3$) δ 171.6 (C^{10}), 156.0 (C^{20}), 155.9 (C^3), 143.9 (C^{23}), 141.3 (C^{28}), 137.9 (C^{17}), 127.7 (C^{26}), 127.1 (C^{27}), 125.1 (C^{25}), 120.0 (C^{24}), 115.2 (C^{18}), 80.6 (C^{12}), 79.2 (C^2), 74.2 (C^{13}), 67.1 (C^{11}), 64.8 (C^{21}), 53.6 (C^9), 47.2 (C^{22}), 40.1 (C^5), 33.8 (C^{14}), 33.0 (C^{16}), 32.0 (C^8), 29.6 (C^6), 28.4 (C^1), 24.0 (C^{15}), 22.2 (C^7); $[\alpha]_D^{26}$ = −21.3 (c = 1, $CHCl_3$); FTIR (neat, υ_{max}, cm^{-1}) 3676 (w), 3297 (b), 2973 (m), 2366 (w), 2169 (w), 1699 (s), 1518 (m), 1451 (w), 1250 (s), 1172 (s), 1066 (m), 1047 (m), 913 (w), 759 (m), 741 (m); R_f 0.36 (7:3 hexanes:EtOAc); m.p. 97–99 °C; HRMS (ESI+) calculated for $C_{34}H_{43}N_2O_6$ $[M+H]^+$ 575.3121, found 575.3138. The diastereomeric excess was determined to be 94% by HPLC analysis. HPLC (Chiralpak AD-H column; 25 cm): 25°C, 210 nm; 1.00 mL/min; 8:2 hexanes/isopropyl alcohol; t_R (*S*,*S*)=11.048 min; t_R (*S*,*R*)=12.686 min.

(*R*)-oct-7-en-1-yn-3-yl *N*[2]-(((9*H*-fluoren-9-yl)methoxy)carbonyl)-*N*[6]-(*tert*-butoxycarbonyl)-*L*-lysinate (2)

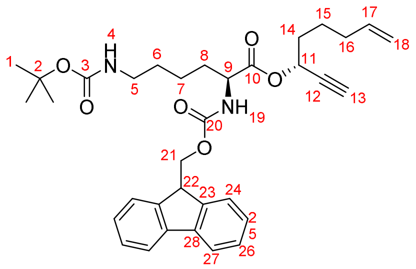

Fmoc-Lys(Boc)-OH (152 mg, 0.325 mmol) was added to a solution of compound **9** (30.0 mg, 0.250 mmol) and PPh_3 (86.0 mg, 0.325mmol) in THF (1.44 mL) and the mixture was cooled to 0 °C. DIAD (64.0 μL, 0.325 mmol) was added dropwise and the reaction mixture was stirred overnight at rt. The solvents were evaporated leaving an off-white residue. Further purification by column chromatography (hexanes:EtOAc, 7:3) gave linker **2** (120 mg, 84%) as an off-white solid.

^{1}H NMR (600 MHz, $CDCl_3$) δ 7.79 (d, *J* = 7.5 Hz, 2H, H^{27}), 7.62 (d, *J* = 7.4 Hz, 2H, H^{24}), 7.42 (t, *J* = 7.5 Hz, 2H, H^{26}), 7.34 (t, *J* = 7.4 Hz, 2H, H^{25}), 5.78 (ddt, *J* = 16.9, 10.2, 6.7 Hz, 1H, H^{17}), 5.46–5.34 (m, *J* = 5.4 Hz, 2H, H^{19}, H^{11}), 5.03 (dd, *J* = 17.1, 1.4 Hz, 1H, H^{18}-*trans*), 4.98 (dd, *J* = 10.2, 1.4 Hz, 1H, H^{18}-*cis*), 4.57 (s, 1H, H^4), 4.47–4.36 (m, 3H, H^9, H^{21}), 4.24 (t, *J* = 7.0 Hz, 1H, H^{22}), 3.18–3.07 (m, 2H, H^5), 2.51 (s, 1H, H^{13}), 2.09 (dt, *J* = 16.7, 8.3 Hz, 2H, H^{16}), 1.95–1.68 (m, 4H, H^8, H^{14}), 1.65–1.50 (m, 6H, H^6, H^7, H^{15}), 1.45 (s, 9H, H^1); ^{13}C NMR (150 MHz, $CDCl_3$) δ 171.3 (C^{10}), 156.1 (C^{20}), 155.9 (C^3), 143.9 (C^{23}), 141.3 (C^{28}), 137.8 (C^{17}), 127.7 (C^{26}), 127.1 (C^{27}), 125.1 (C^{25}), 120.0 (C^{24}), 115.3 (C^{18}), 80.4 (C^{12}), 79.2 (C^2), 74.4 (C^{13}), 67.1 (C^{11}), 64.8 (C^{21}), 53.9 (C^9), 47.2 (C^{22}), 40.1 (C^5), 33.8 (C^{14}), 33.0 (C^{16}), 32.1 (C^8), 29.6 (C^6), 28.4 (C^1), 24.0 (C^{15}), 22.3 (C^7); $[\alpha]_D^{27}$ = +15.7 (c = 1, $CHCl_3$); FTIR (neat, υ_{max}, cm^{-1}) 3676 (w), 3297 (b), 2973 (m), 1699 (m), 1515 (w), 1451 (w), 1394 (m), 1250 (m), 1172 (w), 1066 (s), 890 (w), 760 (w), 741 (w); R_f 0.35 (7:3 hexanes:EtOAc); m.p. 86–89 °C; HRMS (ESI+) calculated for $C_{34}H_{43}N_2O_6$ $[M+H]^+$ 575.3121, found 575.3105. The diastereomeric excess was determined to be 94% by HPLC analysis. HPLC (Chiralpak AD-H column; cm): 25°C, 210 nm; 1.00 mL/min; 8:2 hexanes/isopropyl alcohol; t_R (*S*,*S*)=11.097 min; t_R (*S*,*R*)=12.705 min.

3. Fluorescence Polarisation Assays

<u>Direct FP:</u>

For direct FP assays, FITC-labelled peptides were dissolved in DMSO as stock solutions and diluted with the assay buffer (phosphate buffered saline, pH 7.4, 2 Mm DTT) to a final concentration of 100 nM. MDM2 (residues 2–125) was prepared to a concentration of 10 μM and serially diluted 2-fold with the assay buffer for a 16-point titration in triplicate. FITC-labelled peptide (100 nM, 20 μL) was then mixed with each serially diluted MDM2 solution (top concentration of 10 μM, 20 μL) in the microplate and incubated at 25 °C for 30 min before the measurement was taken. The following filters were used for measuring the FP signals from FITC: an excitation filter 482–16 nm, a dichroic mirror LP504 and an emission filter 530–40 nm.

Data were analysed on GraphPad Prism 5.0 and the dissociation constant, K_d, with standard deviation was determined using the following equation assuming the ratio between the concentration of the bound and that of the total FITC-labelled peptide is proportional to the fluorescence polarisation change:

$$FP = FP_{min} + (FP_{max} - FP_{min}) \cdot \frac{\left[(L_0 + P_0 + K_d) - \sqrt[2]{(L_0 + P_0 + K_d)^2 - 4L_0P_0}\right]}{2L_0}$$

Where FP is the fluorescence polarisation, FP_{min} is the minimum FP, FP_{max} is the maximum FP, L_0 is the total concentration of TAMRA-labelled peptide, P_0 is the total concentration of protein and K_d is the dissociation constant.

Table S1. Binding affinities for peptides **SP1-βA-F (A/B)** and **SP2-βA-F (B)** determined by direct FP.

Peptide[a]	FP K_i (nM)
**SP1-βA-F (A)**	27.9 ± 3.2
**SP1-βA-F (B)**	10.5 ± 10
**SP2-βA-F (B)**	46.2 ± 5.3

[a] **SP2-βA-F (A)** could not be used in direct FP as not enough peptide was available for the assay.

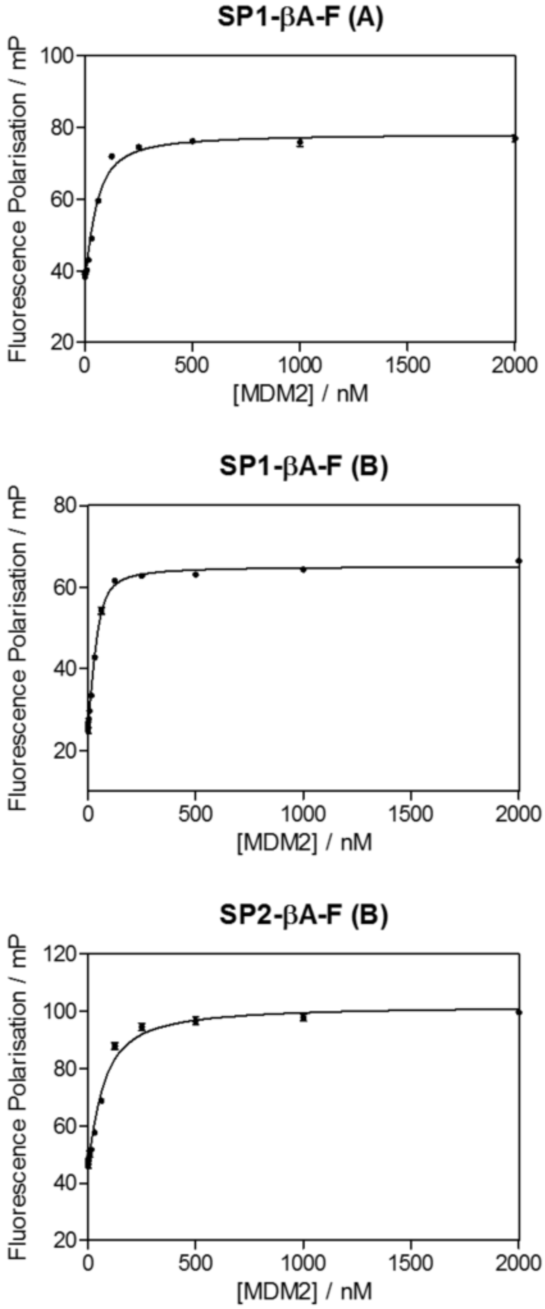

Figure S1. Direct FP titration curve for peptides **SP1-βA-F (A/B)** and **SP2-βA-F (B).**

Competitive FP:

For competitive FP assays, a TAMRA-labelled MDM2-binding peptide (TAMRA-SPT: TAMRA-Ahx-ETF-Orn(N_3)-DLWRLL-Orn(N_3)-EN-NH_2, K_d: 53.8 ± 4.0 nM as measured from direct FP) was used as a tracer and the non-labelled peptides as well as a positive control, nutlin-3a, were used to compete with it for binding to MDM2 protein. A mixture of TAMRA tracer (100 nM) and MDM2 (190 nM) was prepared with the assay buffer. The non-labelled peptides and nutlin-3a were dissolved in DMSO as stock solutions (10 mM) and diluted with PBS to a concentration of 40 μM. Each was further serially diluted 2-fold with the assay for a 16-point titration curve in triplicate. The protein and tracer mixture (final concentration 50 nM, 20 μL) was added to each serially diluted peptide/nutlin-3a (top concentration of 40 μM, 20 μL) in the microplate and incubated at 25 °C for 30 min before the measurement was taken. The following filters were used for measuring the FP signals from TAMRA: an excitation filter 540–20 nm, a dichroic mirror LP 566 nm and an emission filter 590–20 nm.

Data were fitted in GraphPad Prism 5.0 using the following equations described in the literature:[5]

$$a = K_i + K_d + L_0 + (x - P_0)$$
$$b = K_i(L_0 - P_0) + K_d(x - P_0) + K_i K_d$$
$$c = -K_i K_d P_0$$
$$\theta = arc\cos\frac{-2a^3 + 9ab - 27c}{2\sqrt{(a^2 - 3b)^3}}$$
$$F = F_0 + (F_{max} - F_0)\frac{\{2\sqrt{(a^2 - 3b)}\cos(\theta/3) - a\}}{3K_d + \{2\sqrt{(a^2 - 3b)}\cos(\theta/3) - a\}}$$

Where F is the fluorescence polarisation, x is the concentration of the non-labelled peptide or nutlin-3a, K_i is the dissociation constant of the non-labelled peptide or nutlin-3a, K_d is the known dissociation constant of the TAMRA tracer, L_0 is the total concentration of the TAMRA tracer, P_0 is the total concentration of MDM2, F_0 is the fluorescence polarisation when no TAMRA tracer is bound to MDM2 and F_{max} is the fluorescence polarisation when all TAMRA tracer is bound.[6] The unit for K_i is consistent with that of L_0, P_0 and K_d.

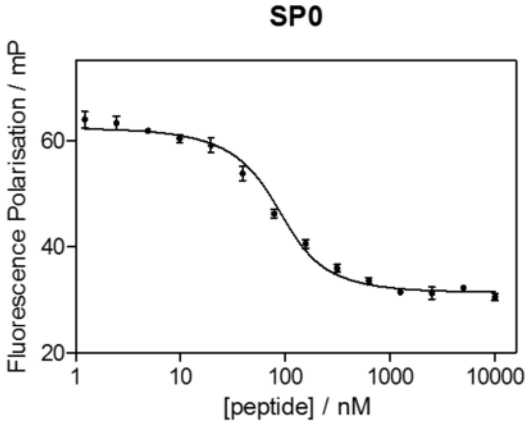

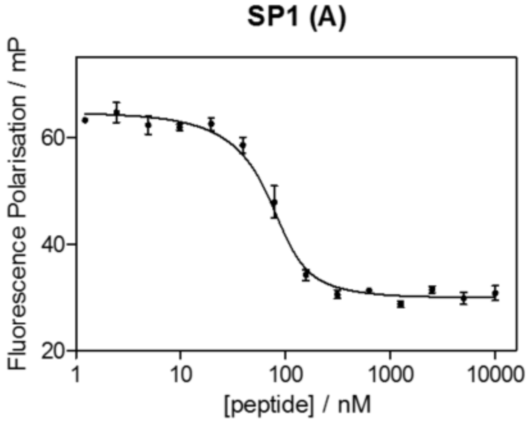

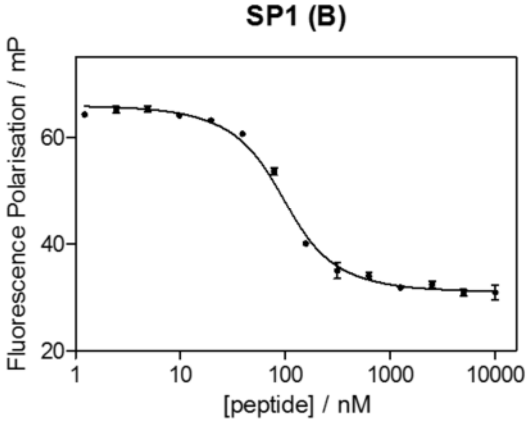

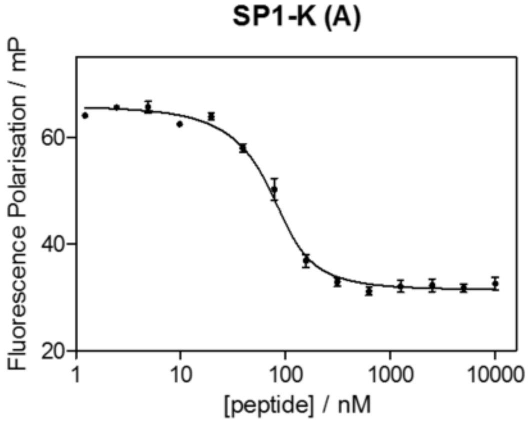

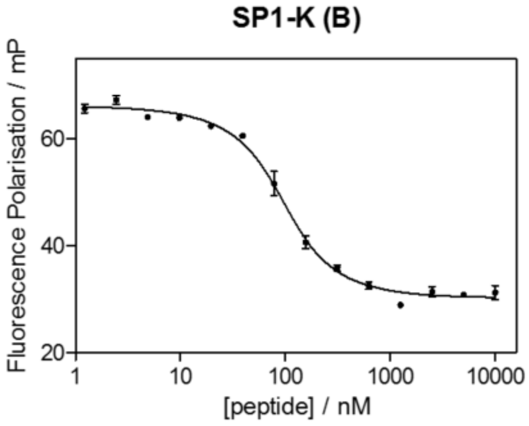

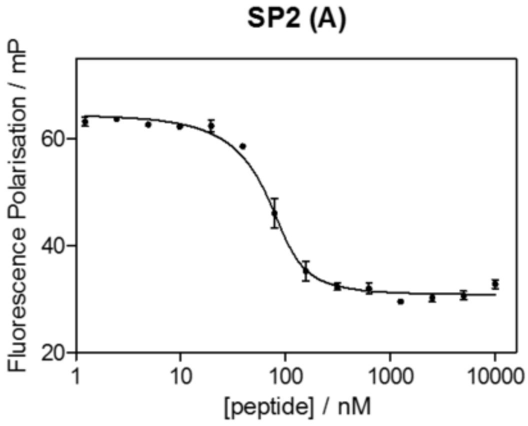

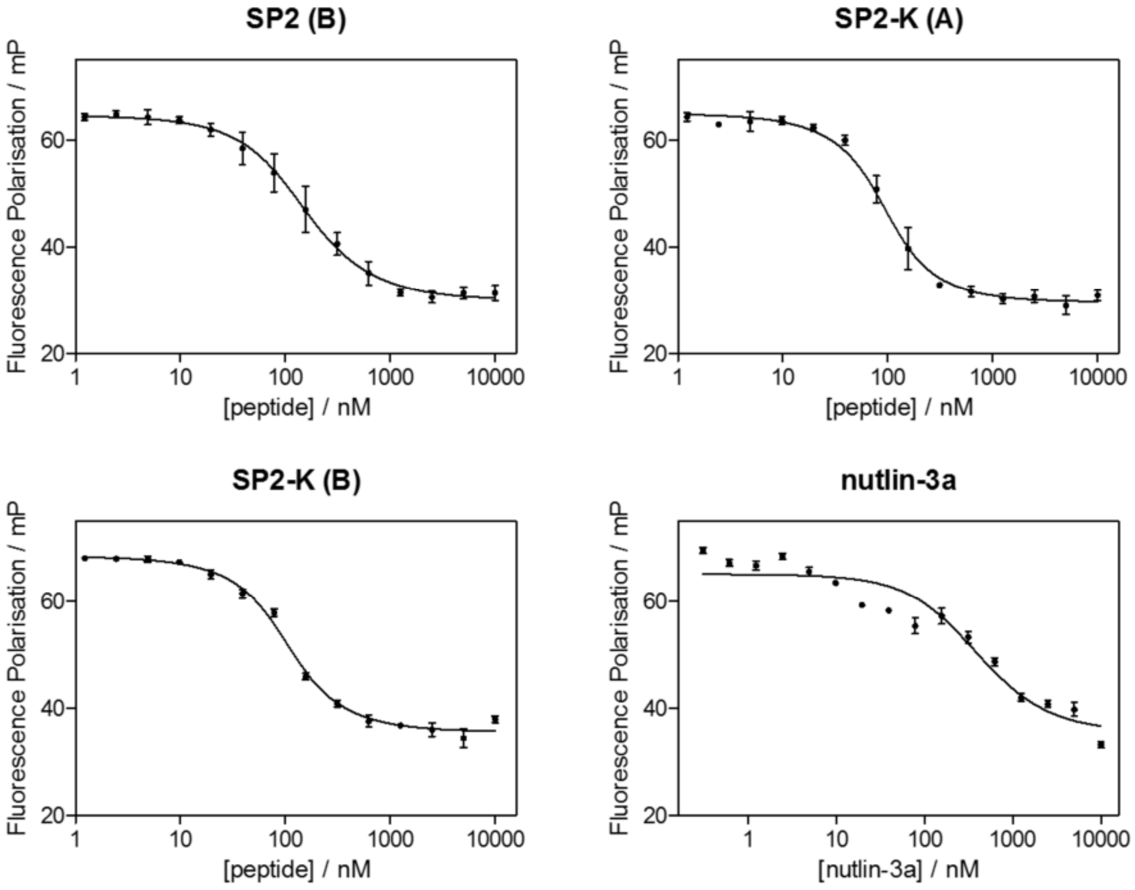

Figure S2. Competitive FP titration curve for peptides and nutlin-3a.

Table S2. IC_{50} values for FP titration curves calculated using Graphpad Prism software using a non-linear fit 4 parameter variable slope analysis. K_d values can be found in Table 1. * nutlin-3a IC_{50} value was generated using a non-linear fit one site analysis as this provided a superior fit to the data points.

Peptide	FP IC_{50} (nM)
**SP0**	71.73 ± 8.56
**SP1 (A)**	80.23 ± 14.99
**SP1 (B)**	105.00 ± 4.20
**SP2 (A)**	69.04 ± 9.95
**SP2 (B)**	131.5 ± 87.90
**SP1-K(A)**	77.06 ± 8.87
**SP1-K (B)**	99.70 ± 6.54
**SP2-K (A)**	93.77 ± 24.20
**SP2-K (B)**	93.99 ± 8.45
**nutlin-3a***	331.03 ± 101.12

The FP tracer peptide was titrated with MDM2 in a direct fluorescence polarisation assay (Figure S3) and the K_d value was calculated to be 53.8 ± 4.0 nM.

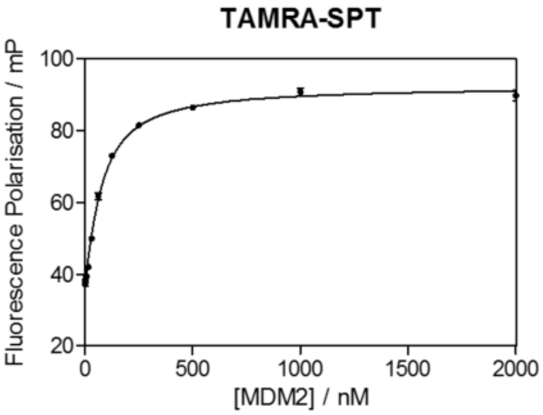

Figure S3. Direct titration of TAMRA-SPT with MDM2.

4. NMR

(*S*)-2-((((9*H*-fluoren-9-yl)methoxy)carbonyl)amino)-4-aminobutanoic acid (3)

^{1}H NMR, 600 MHz, DMSO-d_6

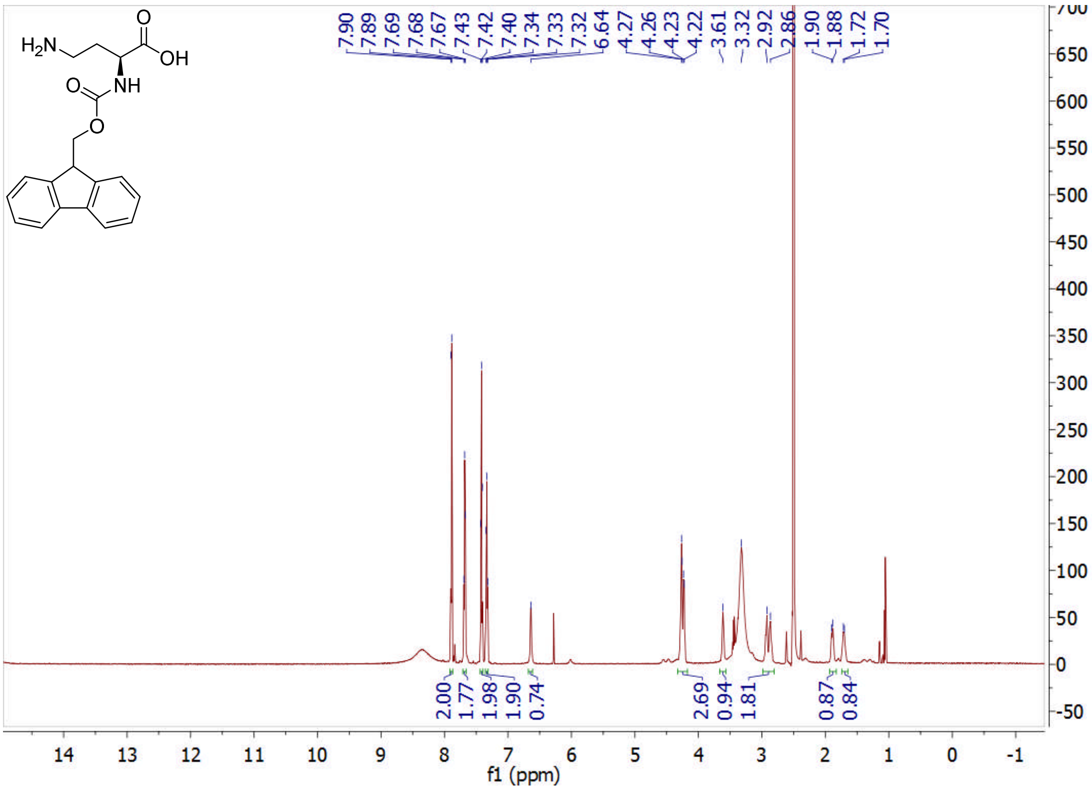

(*S*)-2-((((9*H*-fluoren-9-yl)methoxy)carbonyl)amino)-4-azidobutanoic acid (4)

^{1}H NMR, 600 MHz, $CDCl_3$

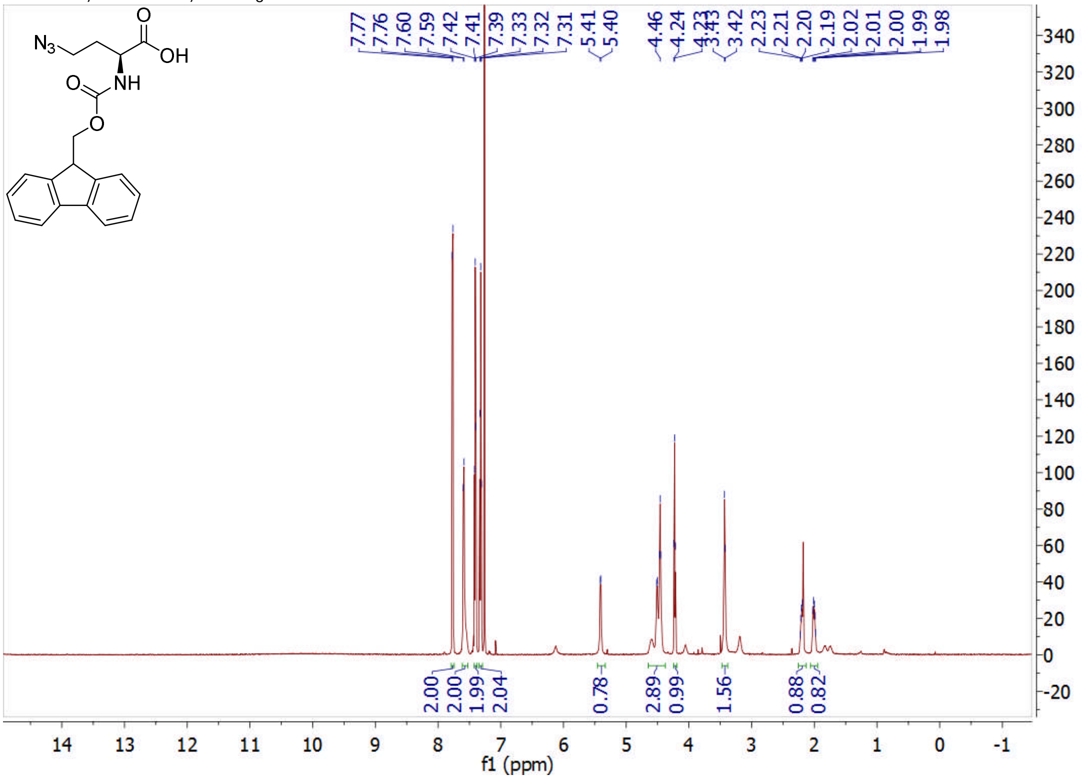

^{13}C NMR, 150 MHz, $CDCl_3$

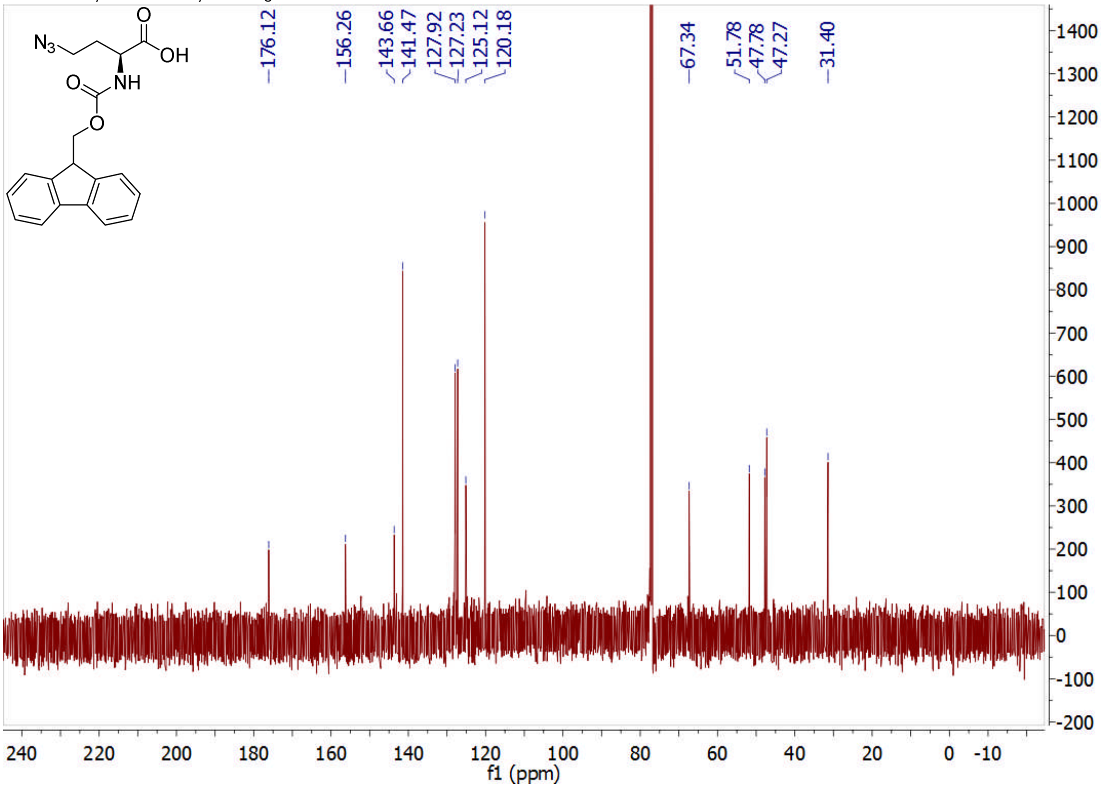

(*E*)-ethyl octa-2,7-dienoate (5)

^{1}H NMR, 600 MHz, $CDCl_3$

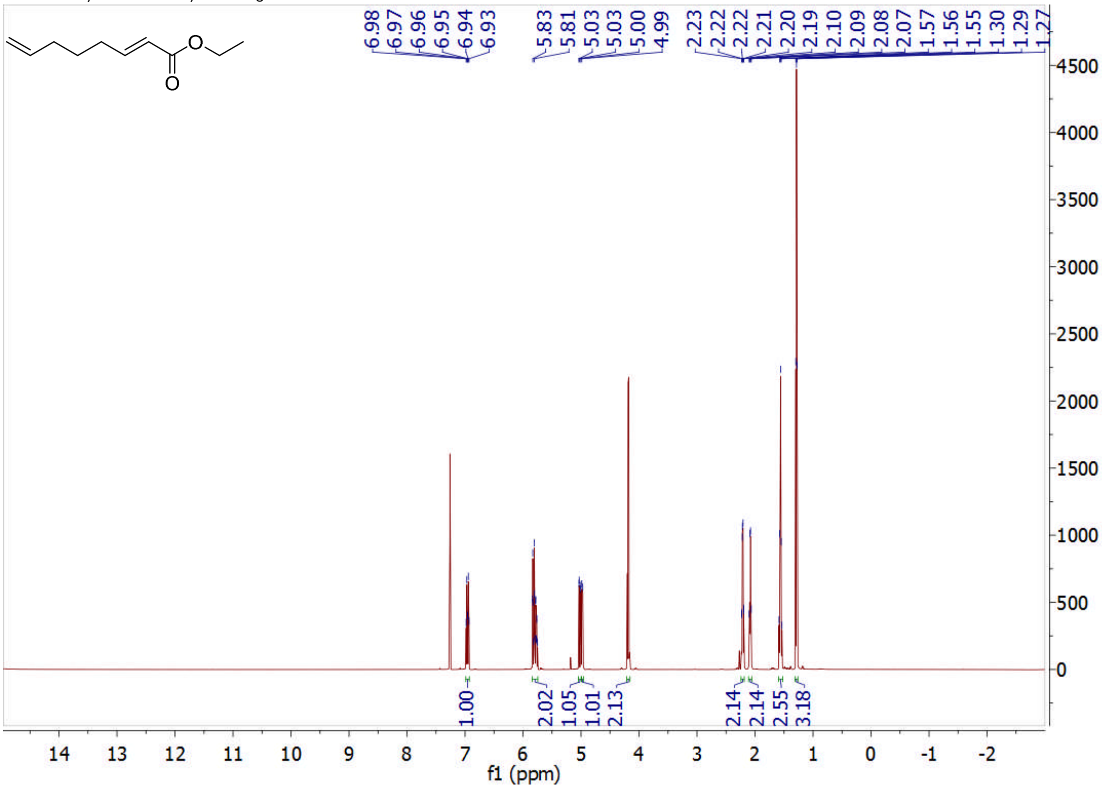

^{13}C NMR, 150 MHz, $CDCl_3$

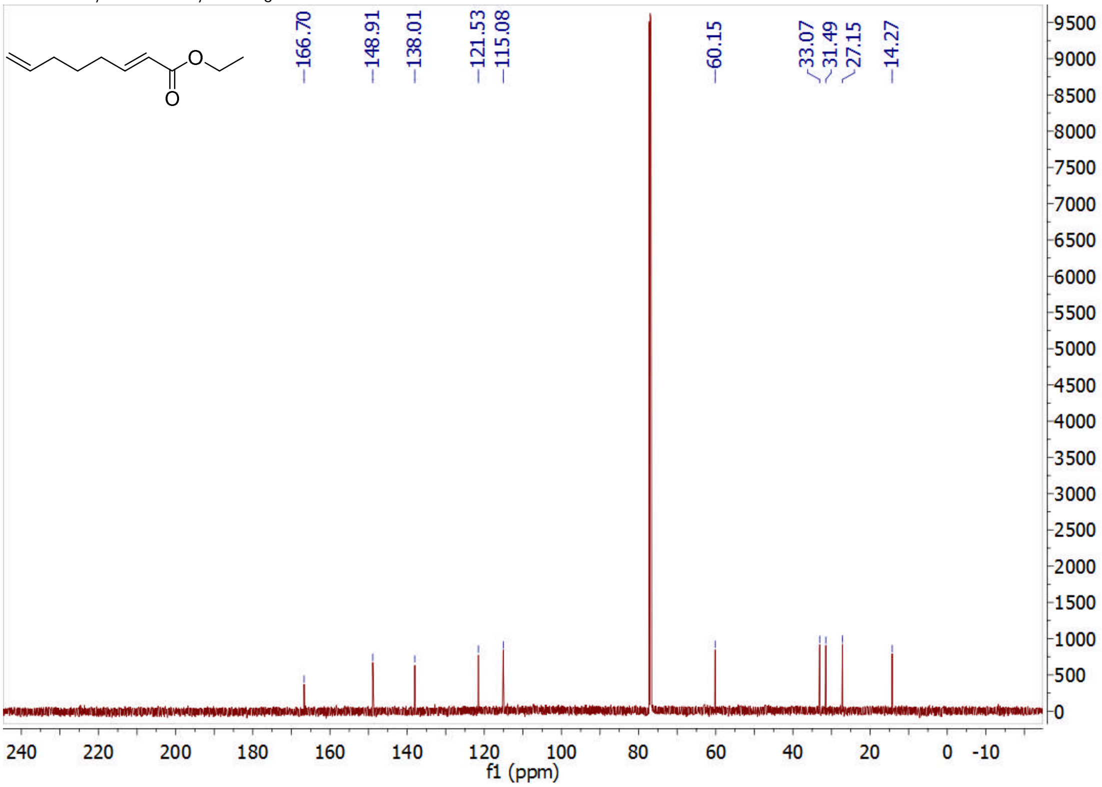

(*E*)-octa-2,7-dien-1-ol (6)

^{1}H NMR, 600 MHz, $CDCl_3$

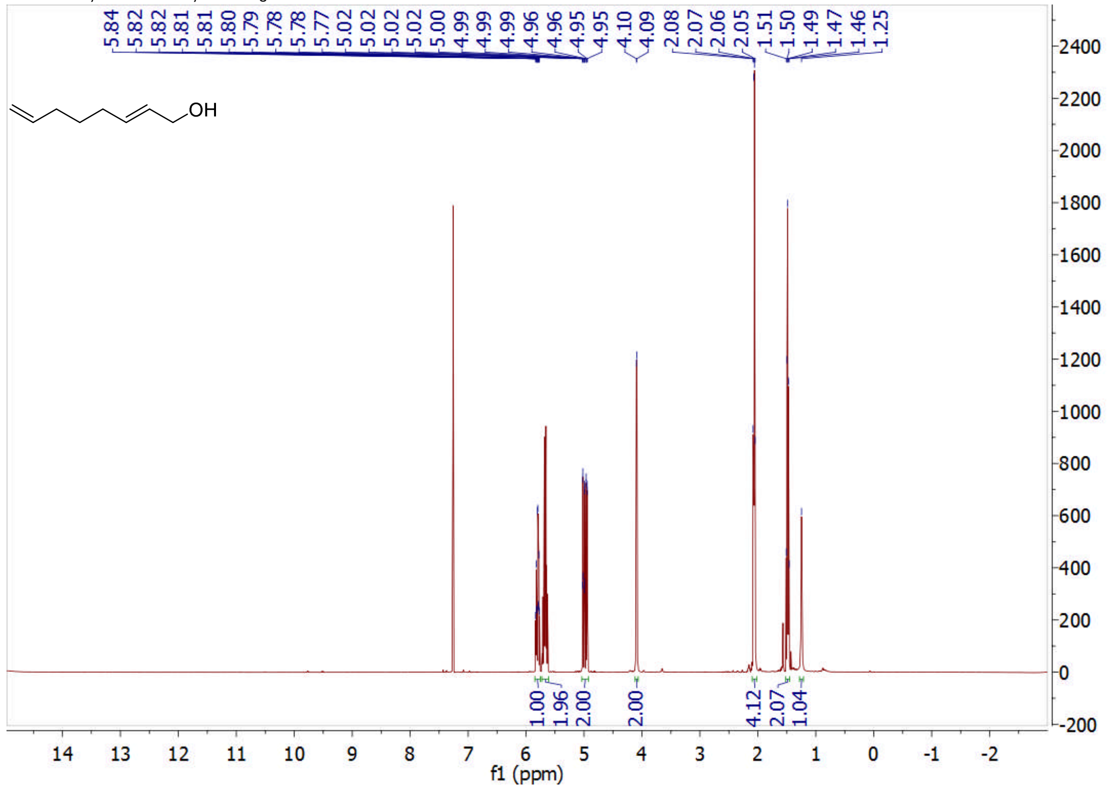

^{13}C NMR, 150 MHz, $CDCl_3$

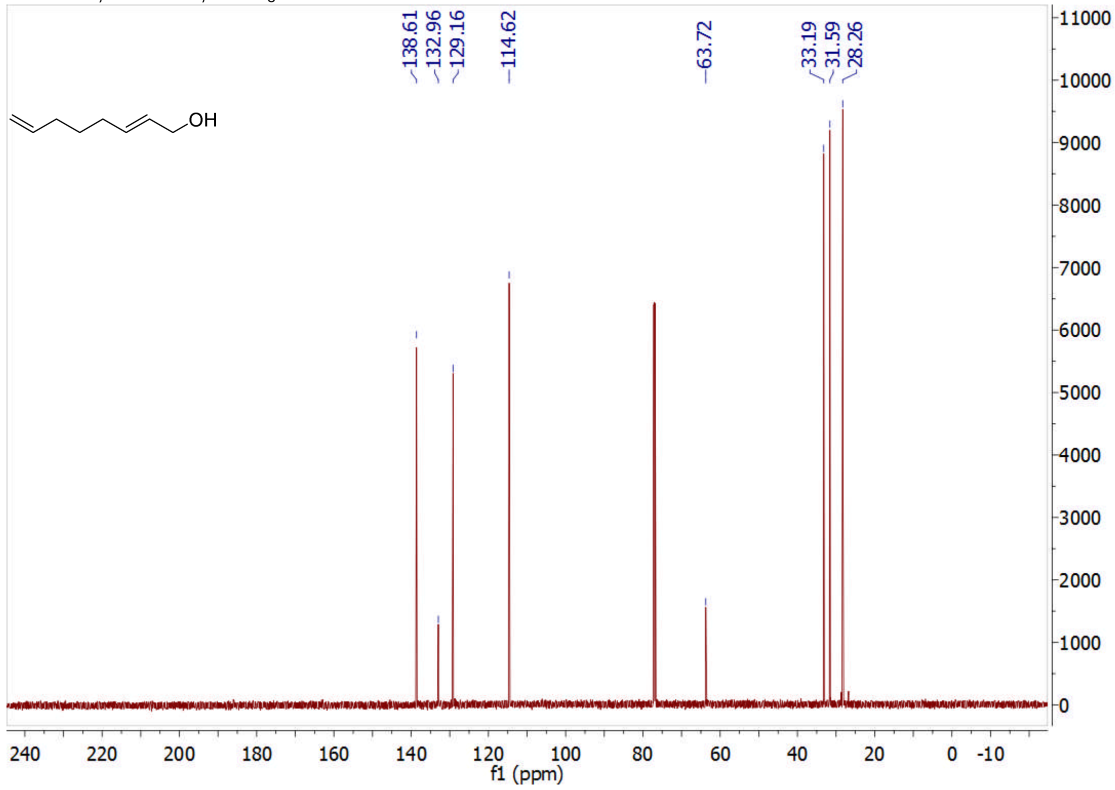

(2*S*,3*S*)-2,3-epoxy-7-octen-1-ol (7)

^{1}H NMR, 600 MHz, $CDCl_3$

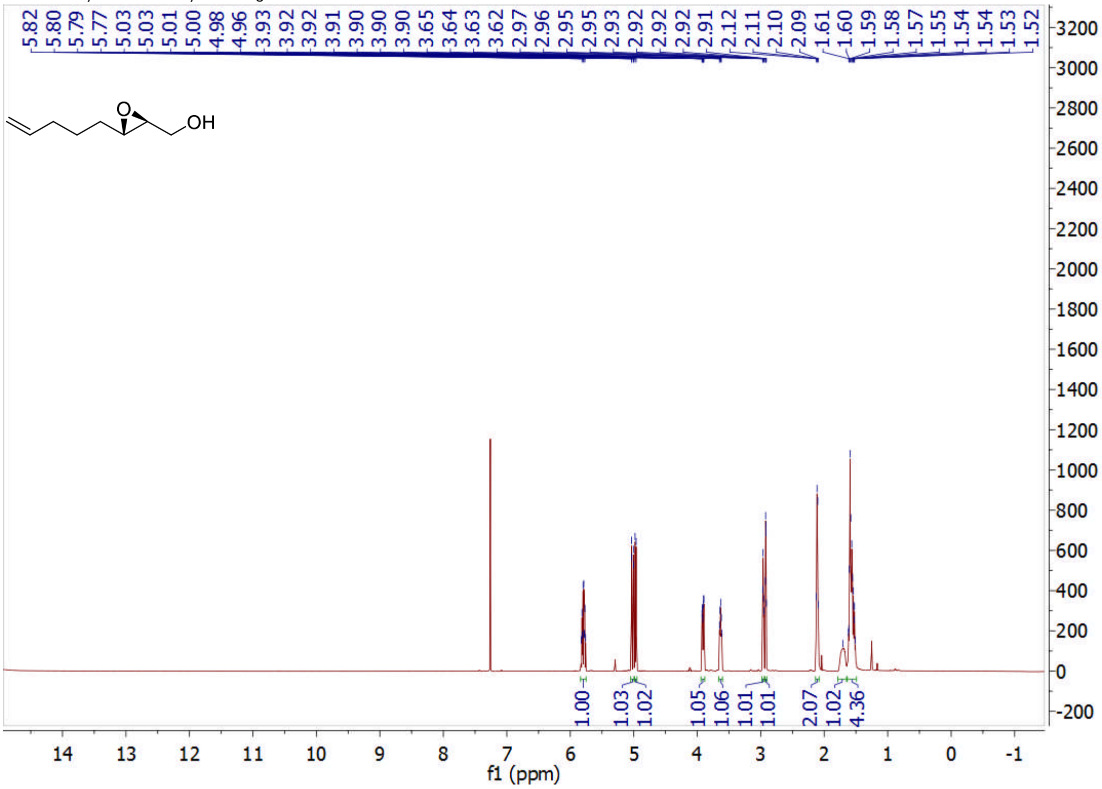

^{13}C NMR, 150 MHz, $CDCl_3$

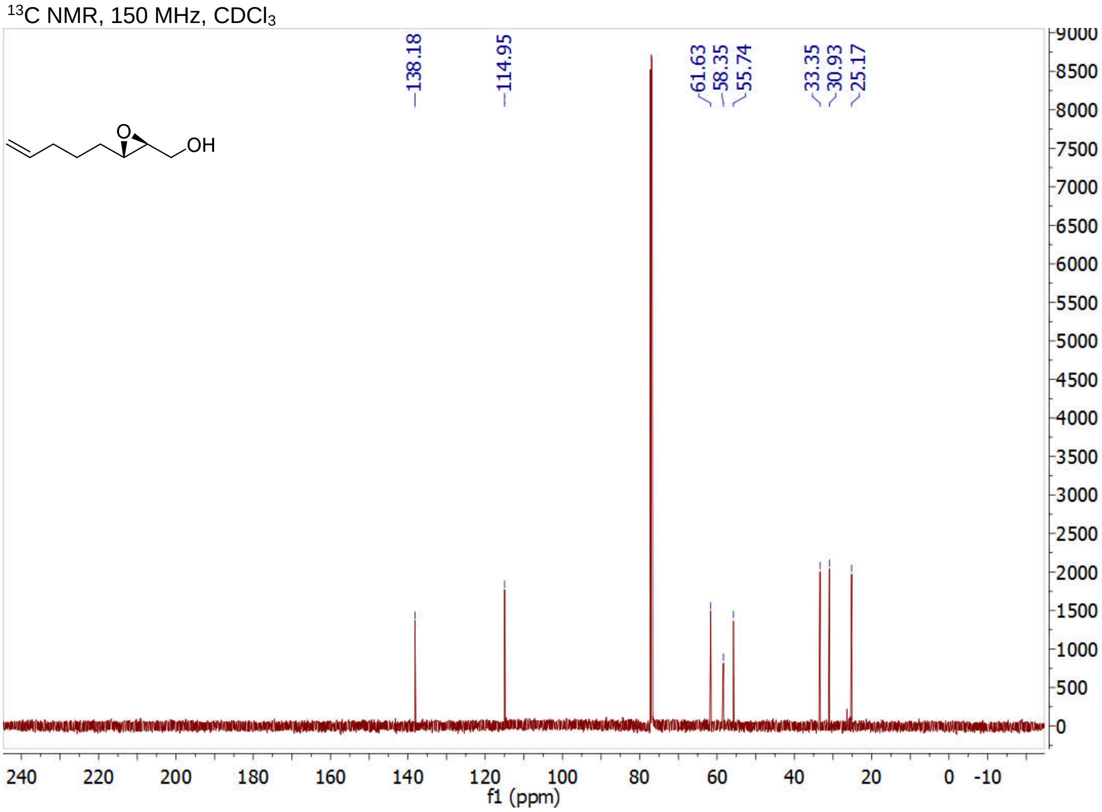

(2*R*,3*S*)-2-(chloromethyl)-3-(pent-4-en-1-yl)oxirane (8)

^{1}H NMR, 600 MHz, $CDCl_3$

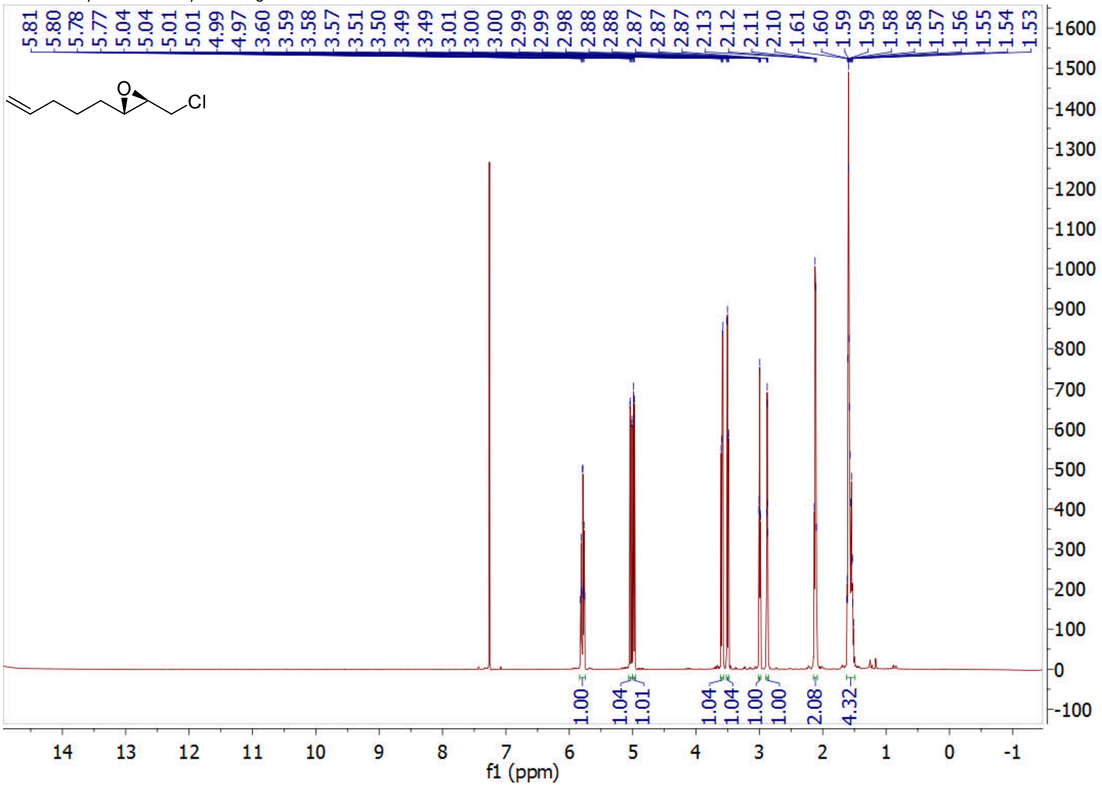

^{13}C NMR, 150 MHz, $CDCl_3$

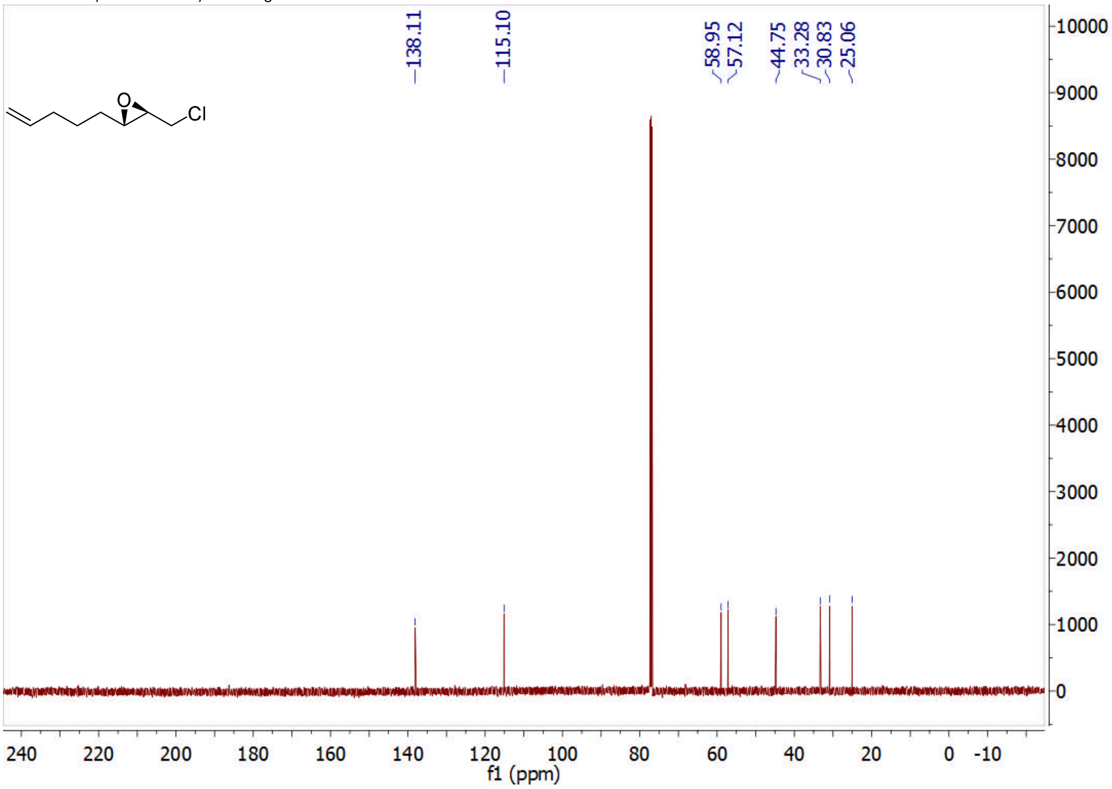

(*S*)-oct-7-en-1-yn-3-ol (9)

^{1}H NMR, 600 MHz, $CDCl_3$

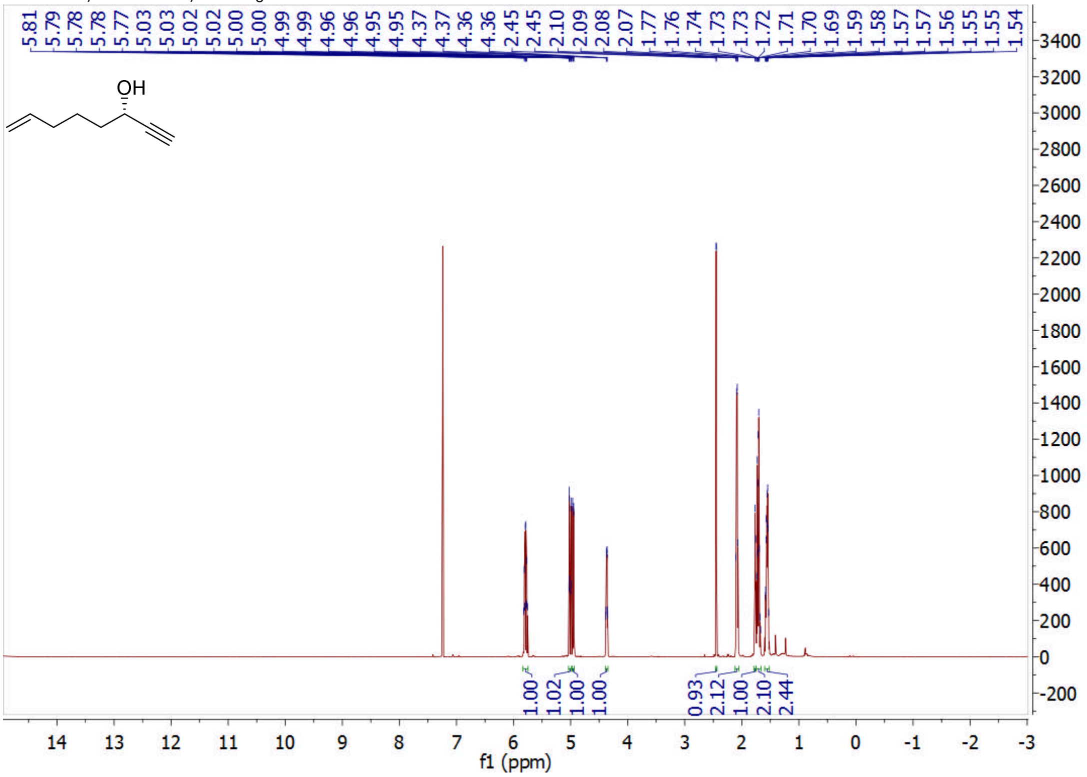

^{13}C NMR, 150 MHz, $CDCl_3$

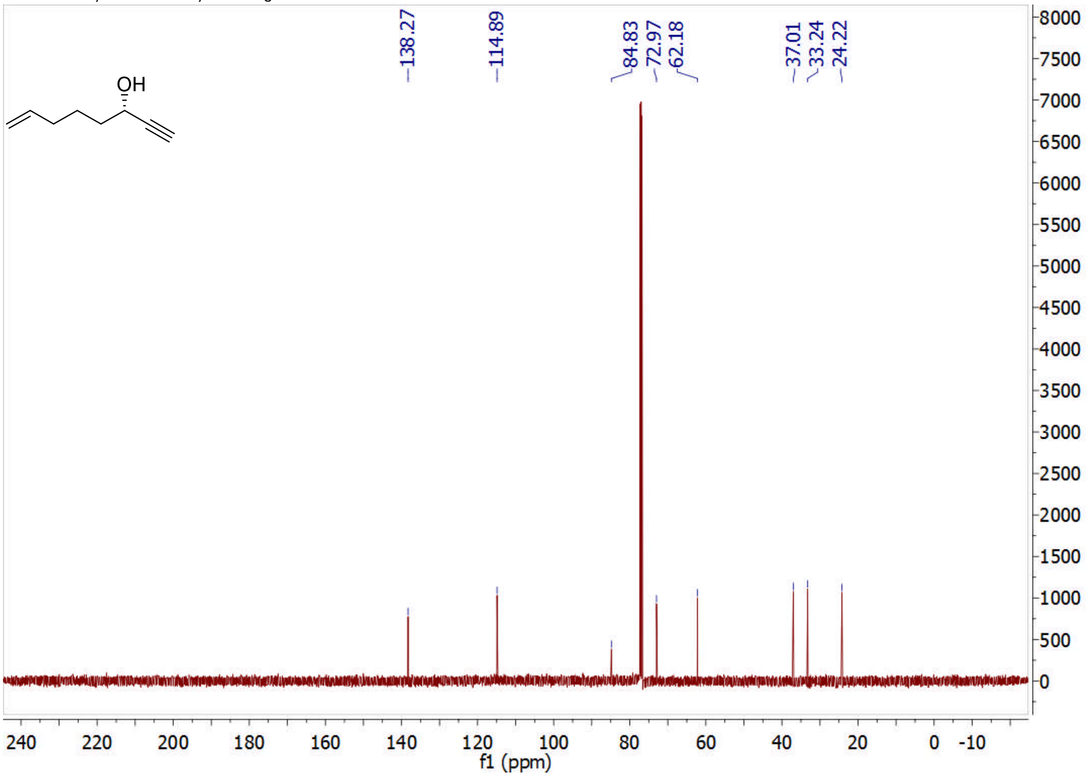

(*S*)-oct-7-en-1-yn-3-yl *N*[2]-(((9*H*-fluoren-9-yl)methoxy)carbonyl)-*N*[6]-(*tert*-butoxycarbonyl)-*L*-lysinate (1)

^{1}H NMR, 600 MHz, $CDCl_3$

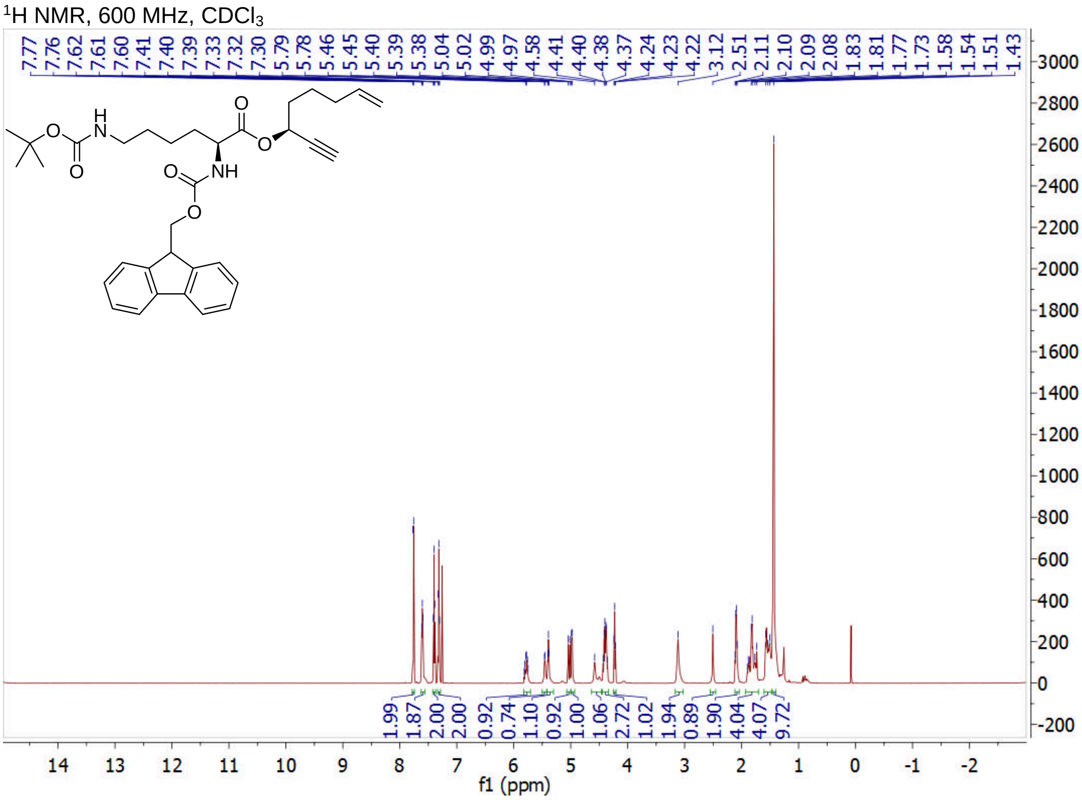

^{13}C NMR, 150 MHz, $CDCl_3$

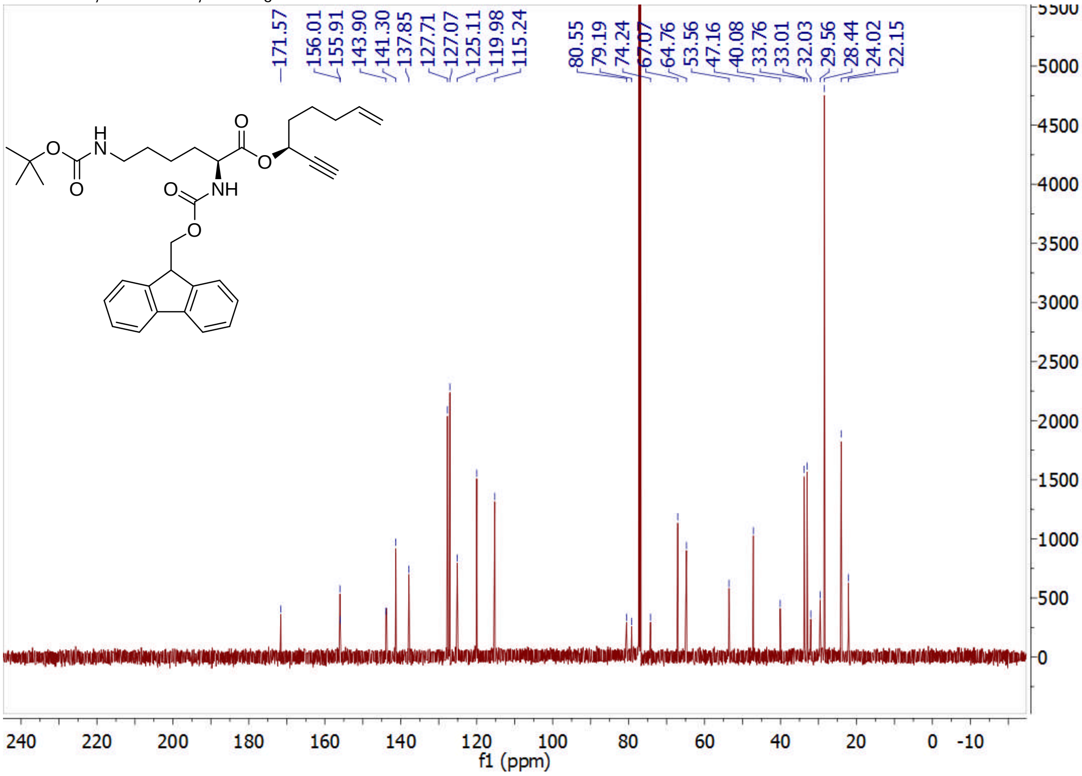

(*R*)-oct-7-en-1-yn-3-yl N^2-(((9*H*-fluoren-9-yl)methoxy)carbonyl)-N^6-(*tert*-butoxycarbonyl)-*L*-lysinate (2)

^{1}H NMR, 600 MHz, $CDCl_3$

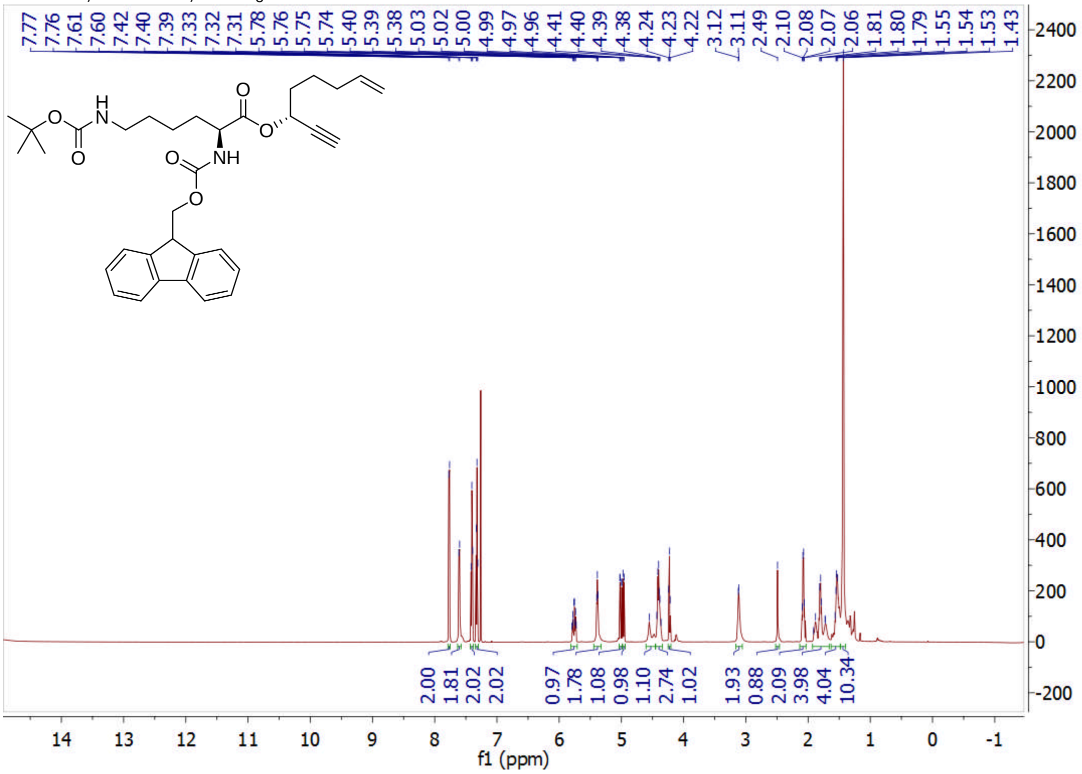

^{13}C NMR, 150 MHz, $CDCl_3$

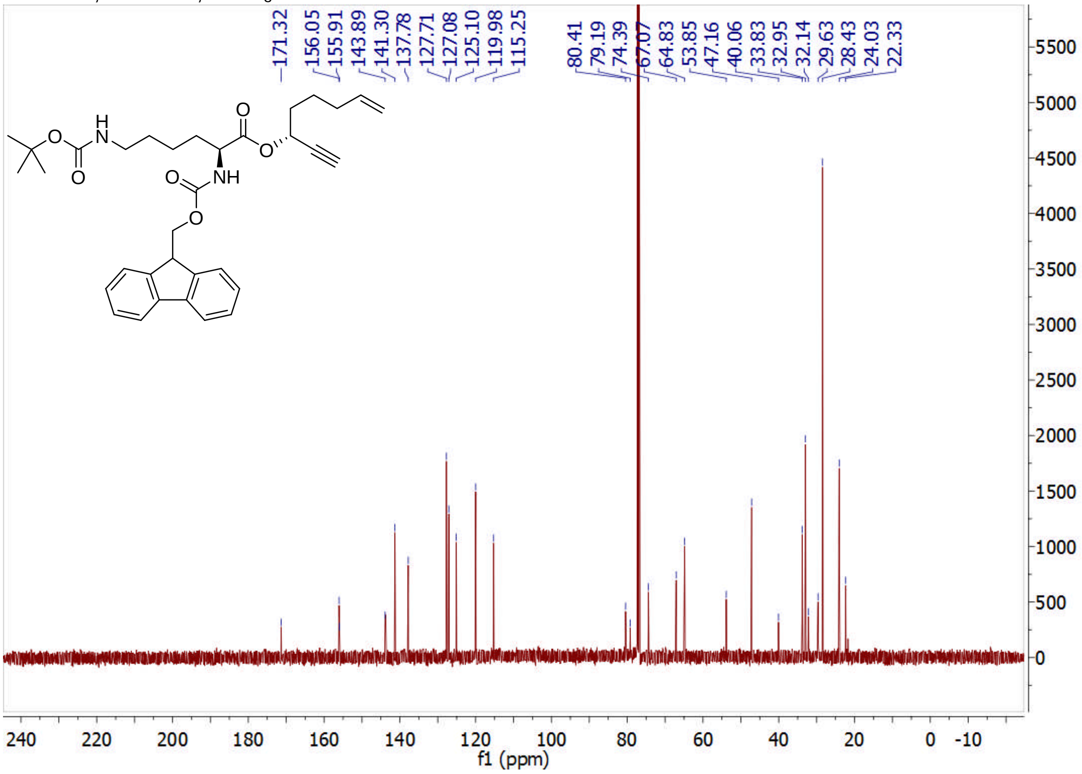

5. Peptide Characterisation

5.1 Peptide HRMS and Analytical HPLC Data

Table S3. Peptide HRMS Data.

Peptide	Formula	Mass	*m*/*z* found	*m*/*z* calculated
**SP0**	$C_{80}H_{120}N_{22}O_{22}$	1740.89	1741.9061	1741.9020 $[M+H]^+$
**SP1 (A)**	$C_{92}H_{140}N_{24}O_{24}$	1965.05	983.5320	983.5309 $[M+2H]^{2+}$
**SP1 (B)**	$C_{92}H_{140}N_{24}O_{24}$	1965.05	983.5312	983.5309 $[M+2H]^{2+}$
**SP2 (A)**	$C_{92}H_{140}N_{24}O_{24}$	1965.05	983.5333	983.5309 $[M+2H]^{2+}$
**SP2 (B)**	$C_{92}H_{140}N_{24}O_{24}$	1965.05	983.5326	983.5309 $[M+2H]^{2+}$
**SP1-K(A)**	$C_{98}H_{152}N_{26}O_{25}$	2093.14	2094.1512	2094.1495 $[M+H]^+$
**SP1-K (B)**	$C_{98}H_{152}N_{26}O_{25}$	2093.14	2094.1511	2094.1495 $[M+H]^+$
**SP2-K (A)**	$C_{98}H_{152}N_{26}O_{25}$	2093.14	2094.1519	2094.1495 $[M+H]^+$
**SP2-K (B)**	$C_{98}H_{152}N_{26}O_{25}$	2093.14	2094.1515	2094.1495 $[M+H]^+$
**SP1-βA-F (A)**	$C_{116}H_{156}N_{26}O_{30}S$	2425.12	2426.1315	2426.1274 $[M+H]^+$
**SP1-βA-F (B)**	$C_{116}H_{156}N_{26}O_{30}S$	2425.12	2426.1311	2426.1274 $[M+H]^+$
**SP2-βA-F (A)**	$C_{116}H_{156}N_{26}O_{30}S$	2425.12	2426.1314	2426.1274 $[M+H]^+$
**SP2-βA-F (B)**	$C_{116}H_{156}N_{26}O_{30}S$	2425.12	2426.1327	2426.1274 $[M+H]^+$

SP0

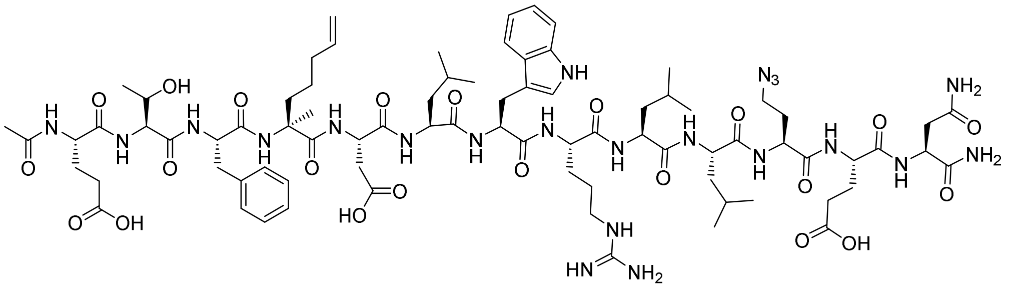

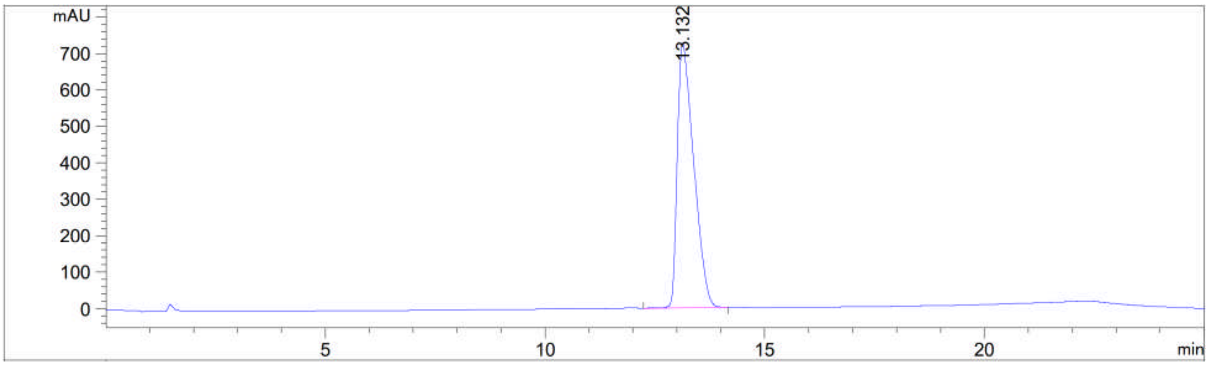

Area Percent Report:

Signal: 220.4 nm
Reference: 360.1

Peak #	Retention Time (min)	Width (min)	Area (mAu × s)	Height (mAu)	Area %
**1**	13.132	0.3867	1.88383×10^4	726.07153	100.0000
**Totals:**			1.88383×10^4	726.07153	

Peak Purity:

Purity factor: 999.942 (128 of 162 spectra exceed the calculated threshold limit.)

Threshold: 999.956

Reference: Peak start/end spectra (integrated) (12.239/14.165)

Spectra: 5

Noise Threshold: 0.089 (12 spectra)

SP1 (A)

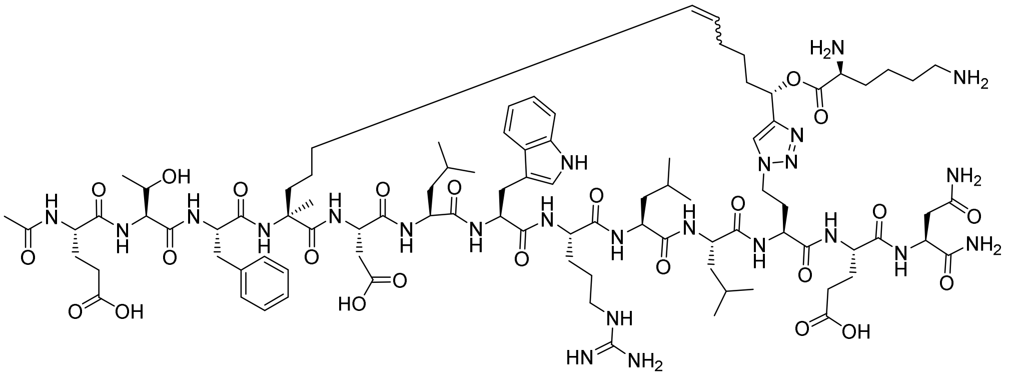

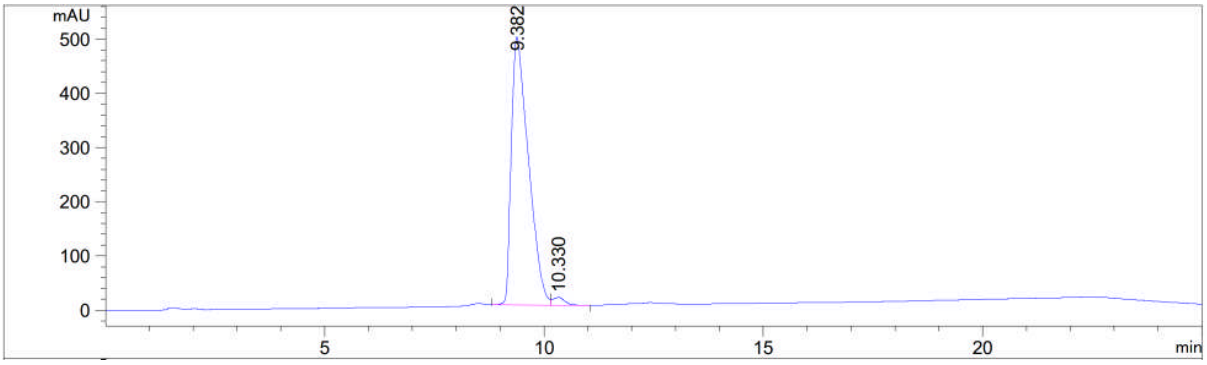

Area Percent Report:

Signal: 220.4 nm
Reference: 360.1

Peak #	Retention Time (min)	Width (min)	Area (mAu × s)	Height (mAu)	Area %
**1**	9.382	0.4162	1.34053×10^4	494.55948	98.0258
**2**	10.330	0.2807	269.97208	14.27432	1.9742
**Totals:**			1.36753×10^4	508.83380	

Peak Purity:

Purity factor: 999.610 (149 of 149 spectra exceed the calculated threshold limit.)

Threshold: 999.899

Reference: Peak start/end spectra (integrated) (8.807/10.167)

Spectra: 5

Noise Threshold: 0.089 (12 spectra)

SP1 (B)

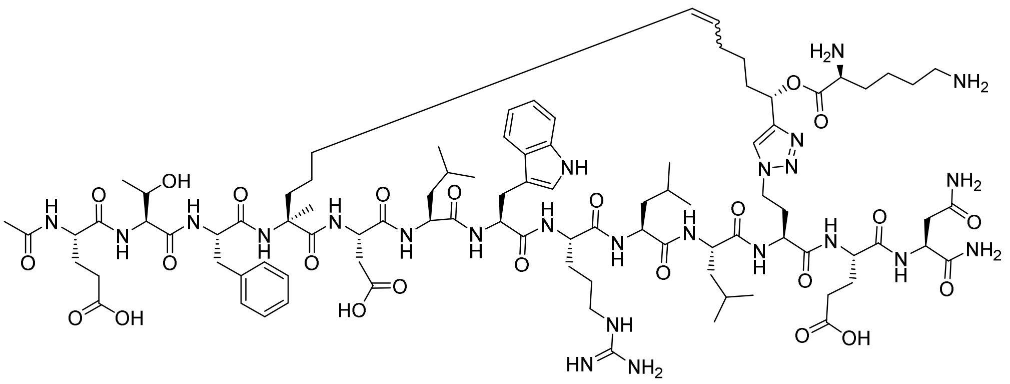

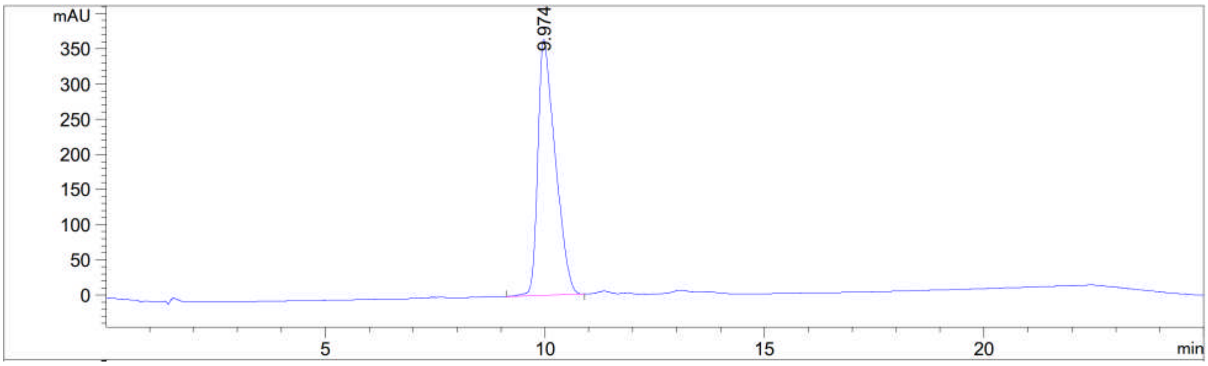

Area Percent Report:

Signal: 220.4 nm
Reference: 360.1

Peak #	Retention Time (min)	Width (min)	Area (mAu × s)	Height (mAu)	Area %
**1**	9.974	0.4170	9998.93848	363.39136	100.0000
**Totals:**			9998.93848	363.39136	

Peak Purity:

Purity factor: 999.789 (103 of 168 spectra exceed the calculated threshold limit.)

Threshold: 999.516

Reference: Peak start/end spectra (integrated) (9.120/10.900)

Spectra: 5

Noise Threshold: 0.108 (12 spectra)

SP2 (A)

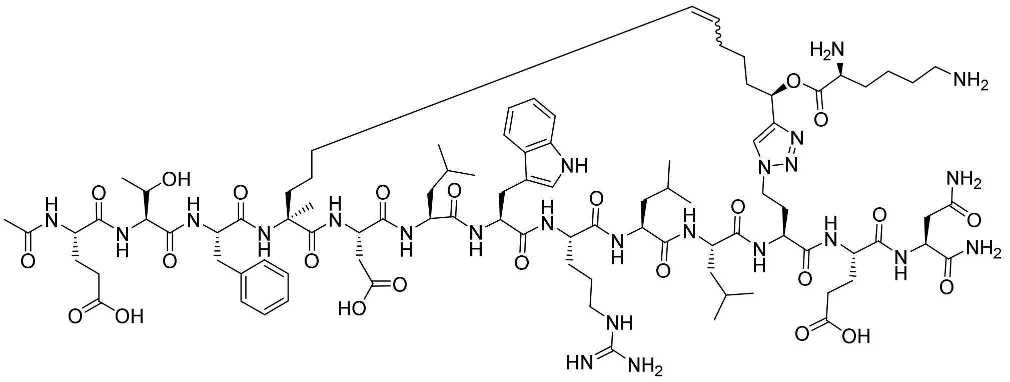

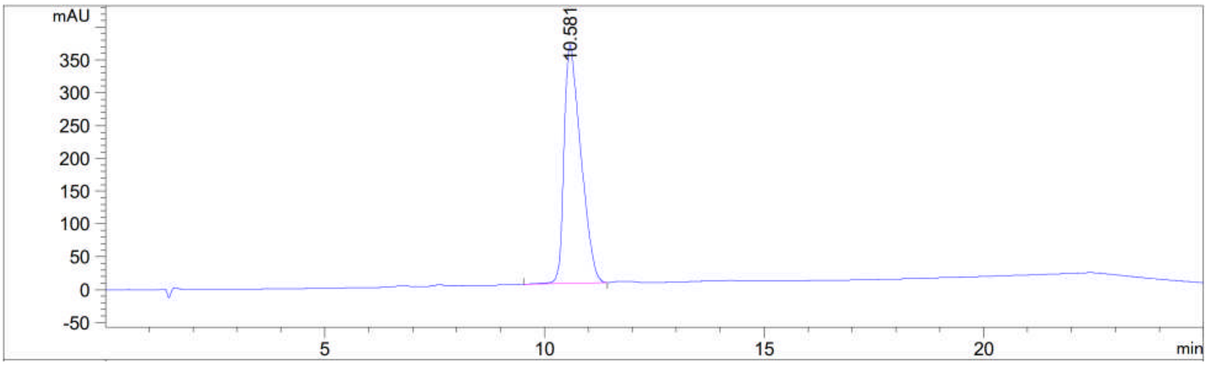

Area Percent Report:

Signal: 220.4 nm
Reference: 360.1

Peak #	Retention Time (min)	Width (min)	Area (mAu × s)	Height (mAu)	Area %
**1**	10.581	0.3994	9653.58203	363.98657	100.0000
**Totals:**			9653.58203	363.98657	

Peak Purity:

Purity factor: 999.202 (153 of 160 spectra exceed the calculated threshold limit.)

Threshold: 999.822

Reference: Peak start/end spectra (integrated) (9.528/11.435)

Spectra: 5

Noise Threshold: 0.067 (12 spectra)

SP2 (B)

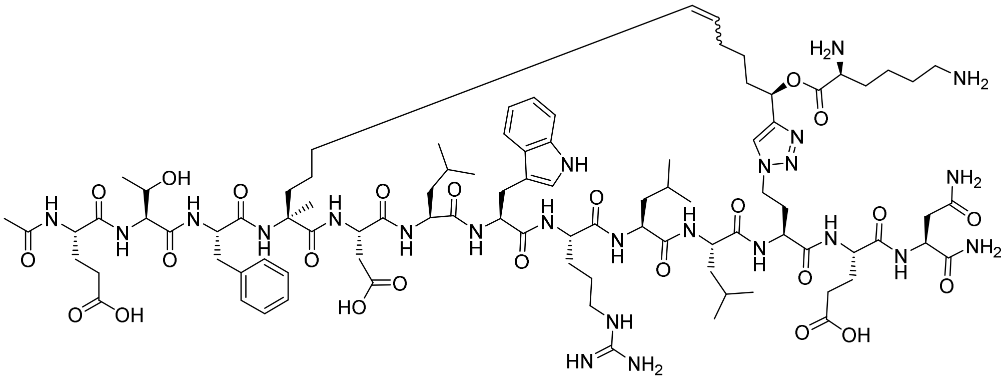

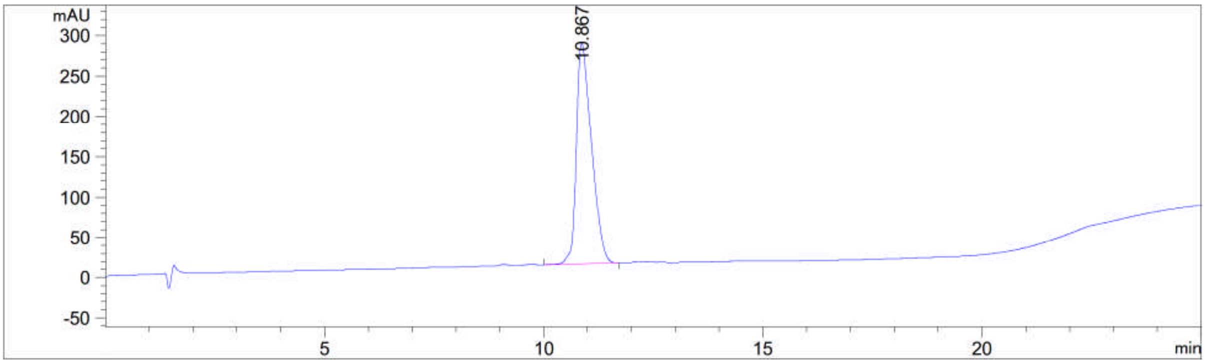

Area Percent Report:

Signal: 220.4 nm
Reference: 360.1

Peak #	Retention Time (min)	Width (min)	Area (mAu × s)	Height (mAu)	Area %
1	10.867	0.3645	6566.1005	273.06116	100.0000
**Totals:**			6566.1005	273.06116	

Peak Purity:

Purity factor: 999.678 (87 of 151 spectra exceed the calculated threshold limit.)

Threshold: 999.912

Reference: Peak start/end spectra (integrated) (10.006/11.719)

Spectra: 5

Noise Threshold: 0.082 (12 spectra)

SP1-K (A)

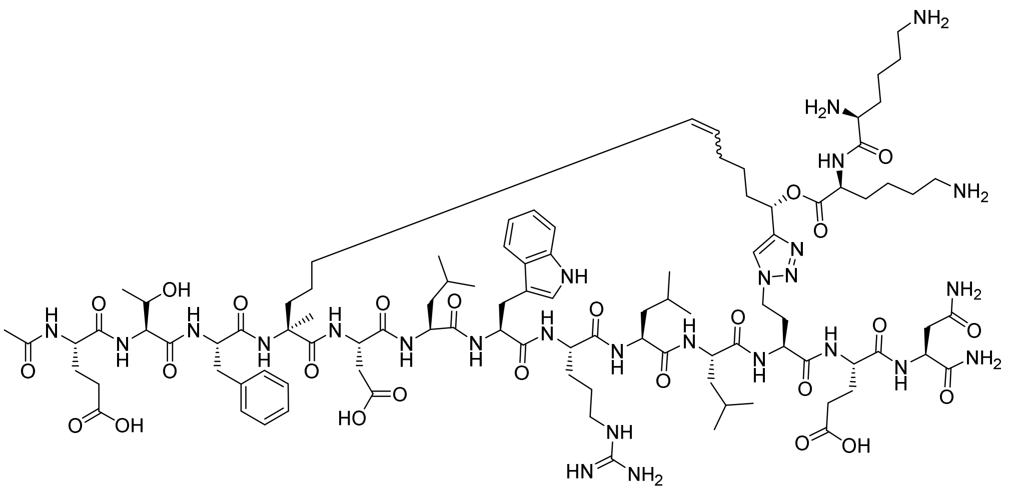

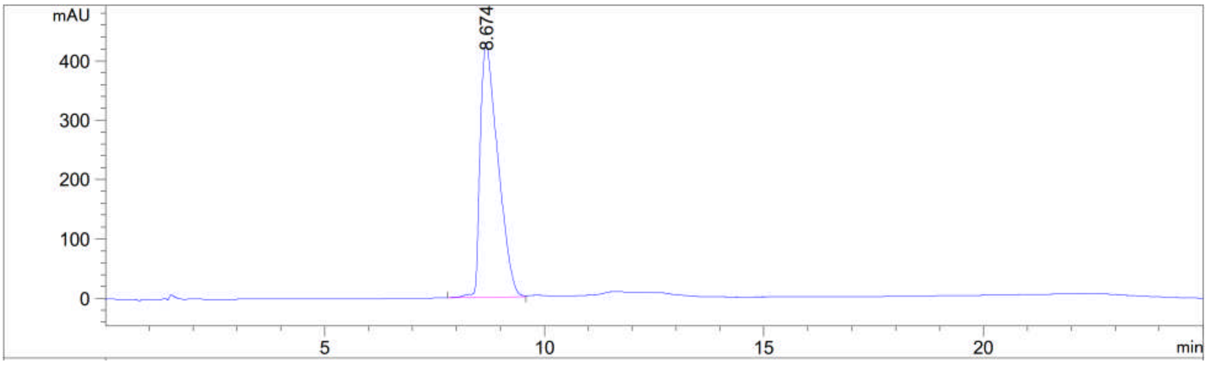

Area Percent Report:

Signal: 220.4 nm
Reference: 360.1

Peak #	Retention Time (min)	Width (min)	Area (mAu × s)	Height (mAu)	Area %
**1**	8.674	0.4192	1.19275×10^4	427.88425	100.0000
**Totals:**			1.19275×10^4	427.88425	

Peak Purity:

Purity factor: 999.616 (156 of 165 spectra exceed the calculated threshold limit.)

Threshold: 999.872

Reference: Peak start/end spectra (integrated) (7.805/9.579)

Spectra: 5

Noise Threshold: 0.054 (12 spectra)

SP1-K (B)

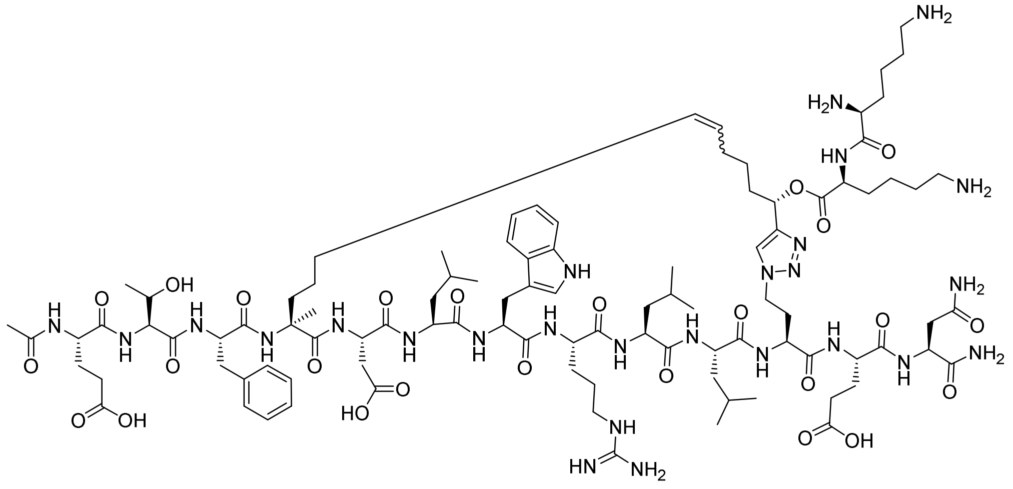

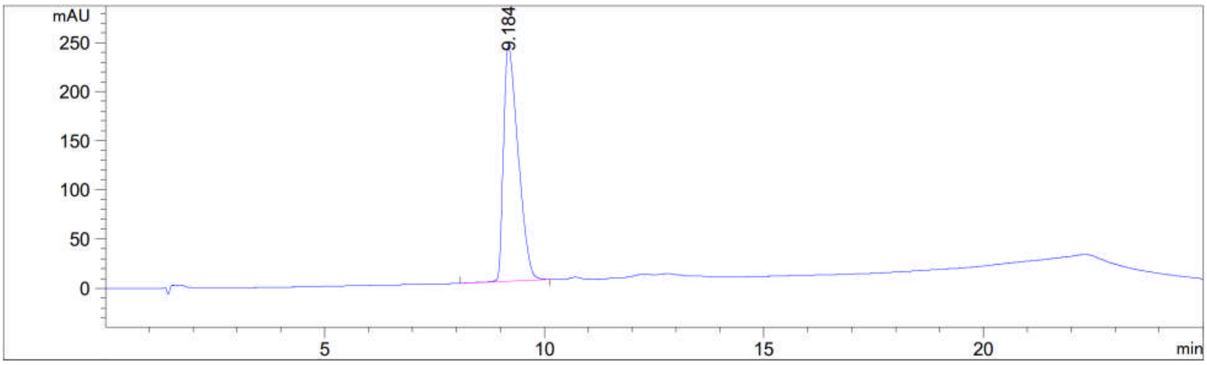

Area Percent Report:

Signal: 220.4 nm
Reference: 360.1

Peak #	Retention Time (min)	Width (min)	Area (mAu × s)	Height (mAu)	Area %
**1**	9.184	0.3584	5524.42285	240.15344	100.0000
**Totals:**			5524.42285	240.15344	

Peak Purity:

Purity factor: 999.943 (95 of 127 spectra exceed the calculated threshold limit.)

Threshold: 999.953

Reference: Peak start/end spectra (integrated) (8.086/10.133)

Spectra: 5

Noise Threshold: 0.054 (12 spectra)

SP2-K (A)

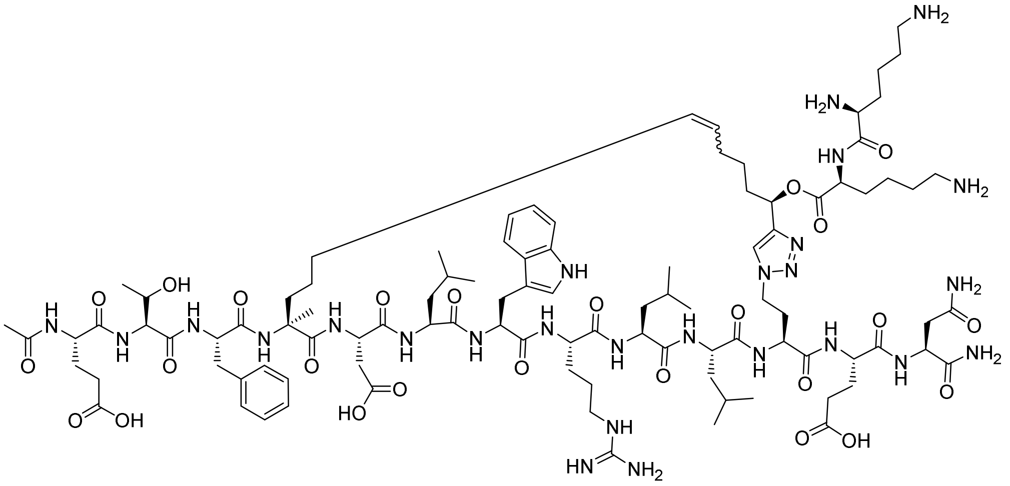

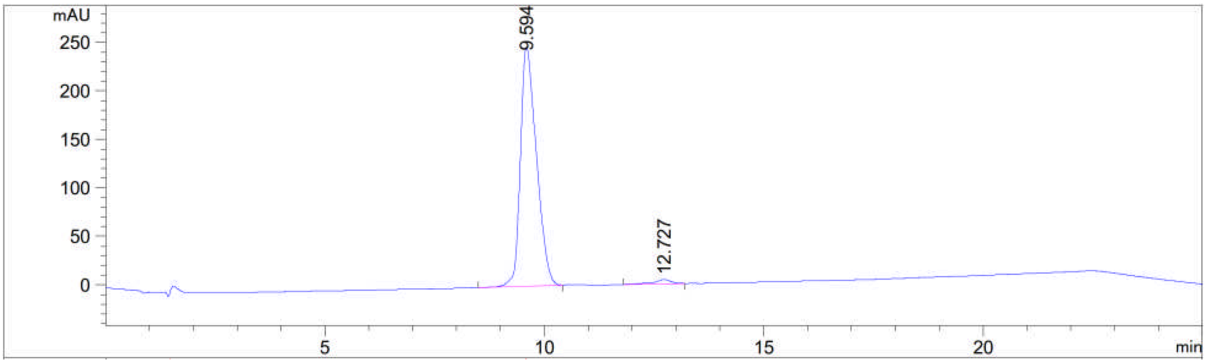

Area Percent Report:

Signal: 220.4 nm
Reference: 360.1

Peak #	Retention Time (min)	Width (min)	Area (mAu × s)	Height (mAu)	Area %
**1**	9.594	0.3867	6268.43604	246.44315	98.1747
**2**	12.727	0.3727	116.54668	4.43340	1.8253
**Totals:**			6384.98271	250.87655	

Peak Purity:

Purity factor: 997.252 (67 of 158 spectra exceed the calculated threshold limit.)

Threshold: 999.541

Reference: Peak start/end spectra (integrated) (8.495/10.422)

Spectra: 5

Noise Threshold: 0.072 (12 spectra)

SP2-K (B)

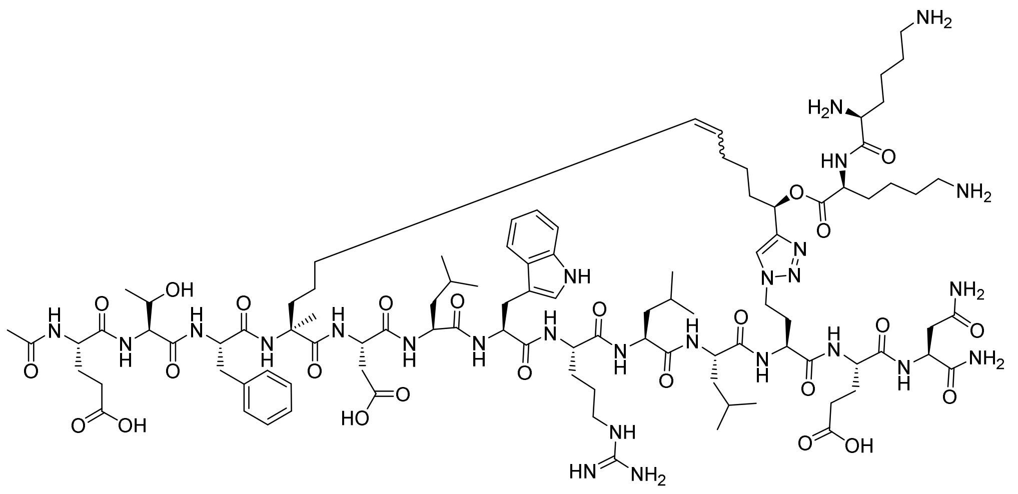

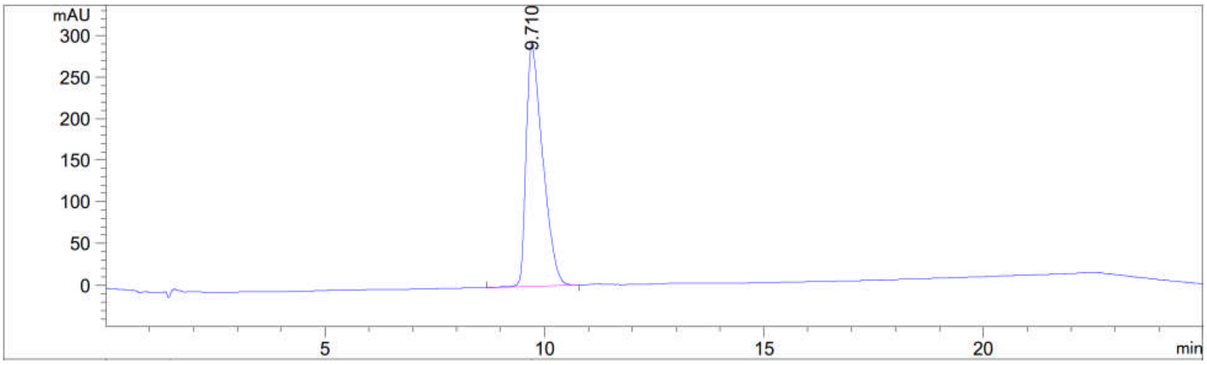

Area Percent Report:

Signal: 220.4 nm
Reference: 360.1

Peak #	Retention Time (min)	Width (min)	Area (mAu × s)	Height (mAu)	Area %
**1**	9.710	0.3825	7572.76514	290.12811	100.0000
**Totals:**			7572.76514	290.12811	

Peak Purity:

Purity factor: 999.803 (75 of 154 spectra exceed the calculated threshold limit.)

Threshold: 999.865

Reference: Peak start/end spectra (integrated) (8.689/10.783)

Spectra: 5

Noise Threshold: 0.076 (12 spectra)

SP1-βA-F (A)

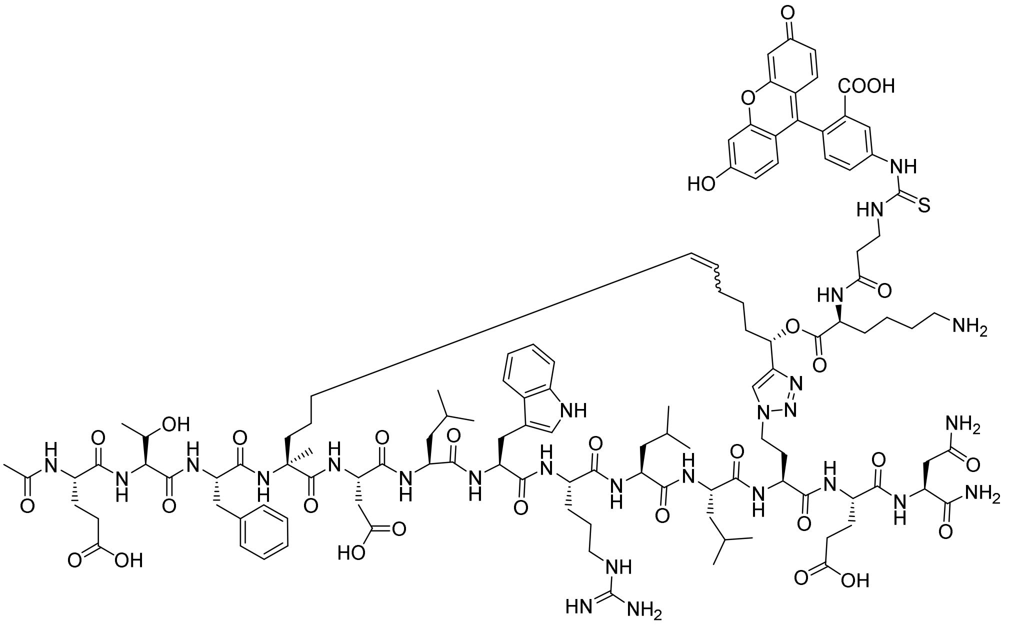

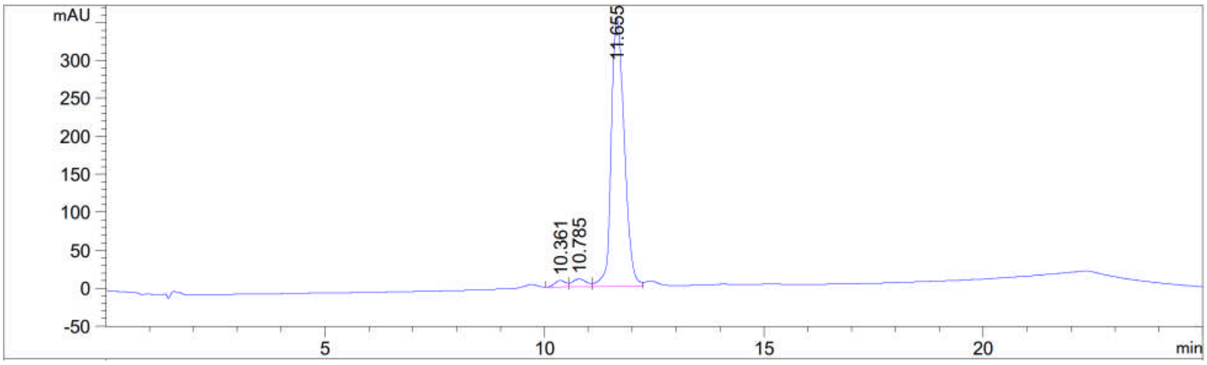

Area Percent Report:

Signal: 220.4 nm
Reference: 360.1

Peak #	Retention Time (min)	Width (min)	Area (mAu × s)	Height (mAu)	Area %
**1**	10.361	0.2660	150.59940	8.61136	1.9628
**2**	10.785	0.3285	234.30444	10.49400	3.0537
**3**	11.655	0.3236	7287.81494	351.89059	94.9835
**Totals:**			7672.71878	370.99595	

Peak Purity:

Purity factor: 997.948 (108 of 128 spectra exceed the calculated threshold limit.)

Threshold: 999.751

Reference: Peak start/end spectra (integrated) (11.092/12.245)

Spectra: 5

Noise Threshold: 0.071 (12 spectra)

SP1-βA-F (B)

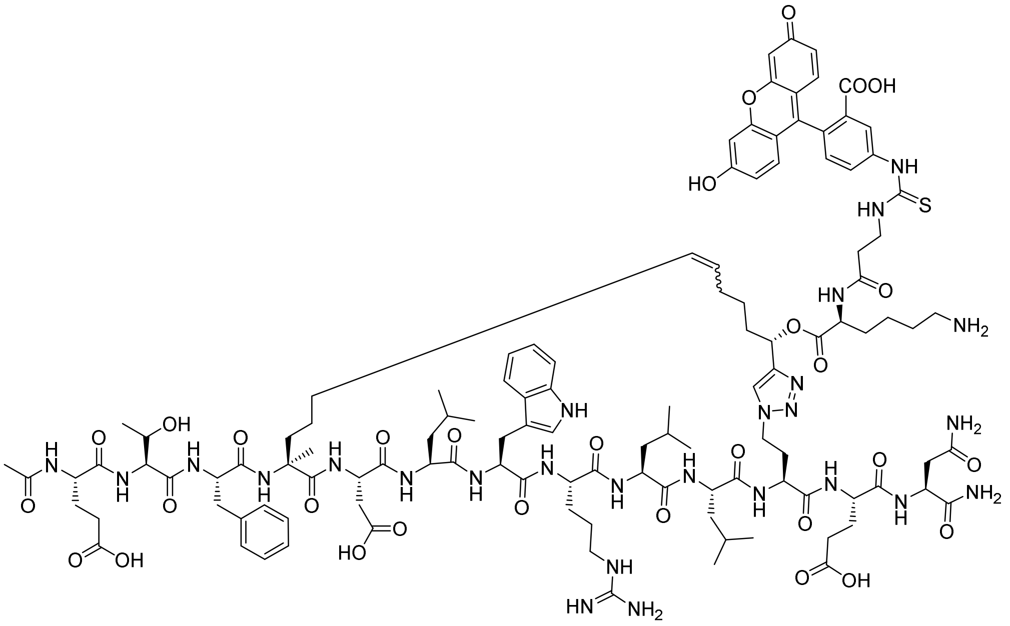

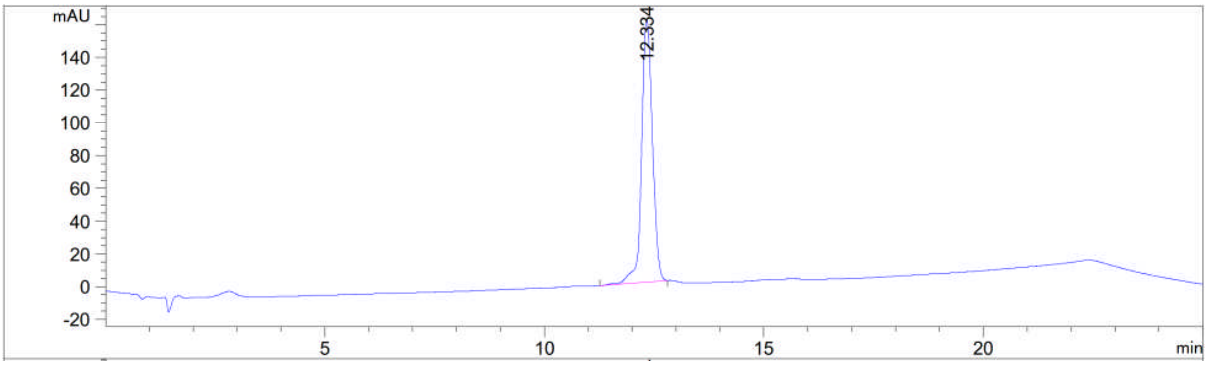

Area Percent Report:

Signal: 220.4 nm
Reference: 360.1

Peak #	Retention Time (min)	Width (min)	Area (mAu × s)	Height (mAu)	Area %
**1**	12.334	0.2694	2761.02124	159.94292	100.0000
**Totals:**			2761.02124	159.94292	

Peak Purity:

Purity factor: 999.334 (72 of 116 spectra exceed the calculated threshold limit.)

Threshold: 999.738

Reference: Peak start/end spectra (integrated) (11.266/12.806)

Spectra: 5

Noise Threshold: 0.049 (12 spectra)

SP2-βA-F (A)

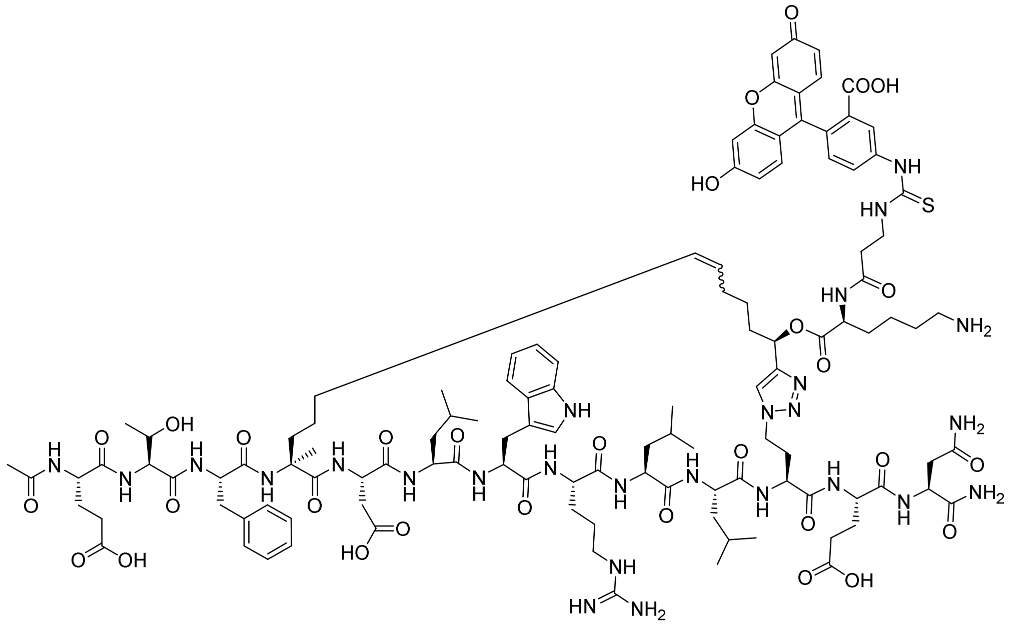

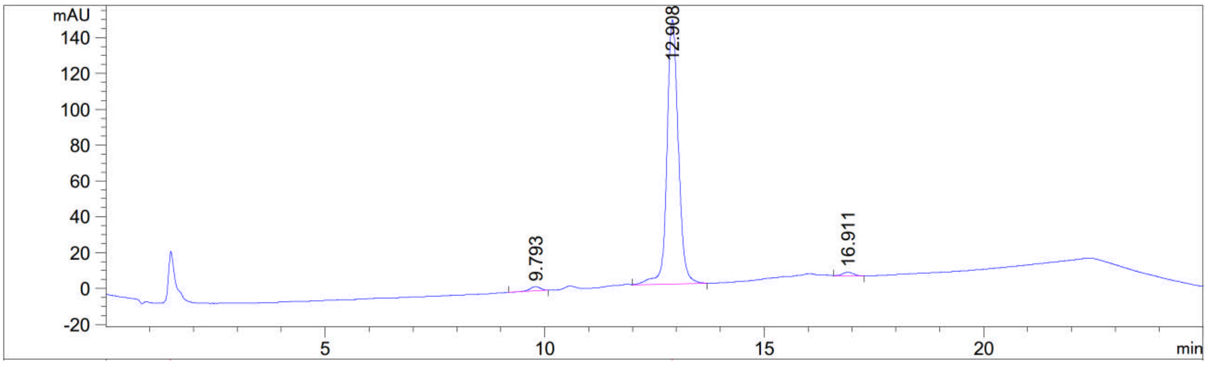

Area Percent Report:

Signal: 220.4 nm
Reference: 360.1

Peak #	Retention Time (min)	Width (min)	Area (mAu × s)	Height (mAu)	Area %
**1**	9.793	0.2560	42.46175	2.42812	1.5549
**2**	12.908	0.2783	2654.98315	147.31358	97.2196
**3**	16.911	0.2412	33.46767	2.02152	1.2255
**Totals:**			2730.91258	151.76322	

Peak Purity:

Purity factor: 999.061 (87 of 105 spectra exceed the calculated threshold limit.)

Threshold: 999.734

Reference: Peak start/end spectra (integrated) (11.988/13.708)

Spectra: 5

Noise Threshold: 0.065 (12 spectra)

SP2-βA-F (B)

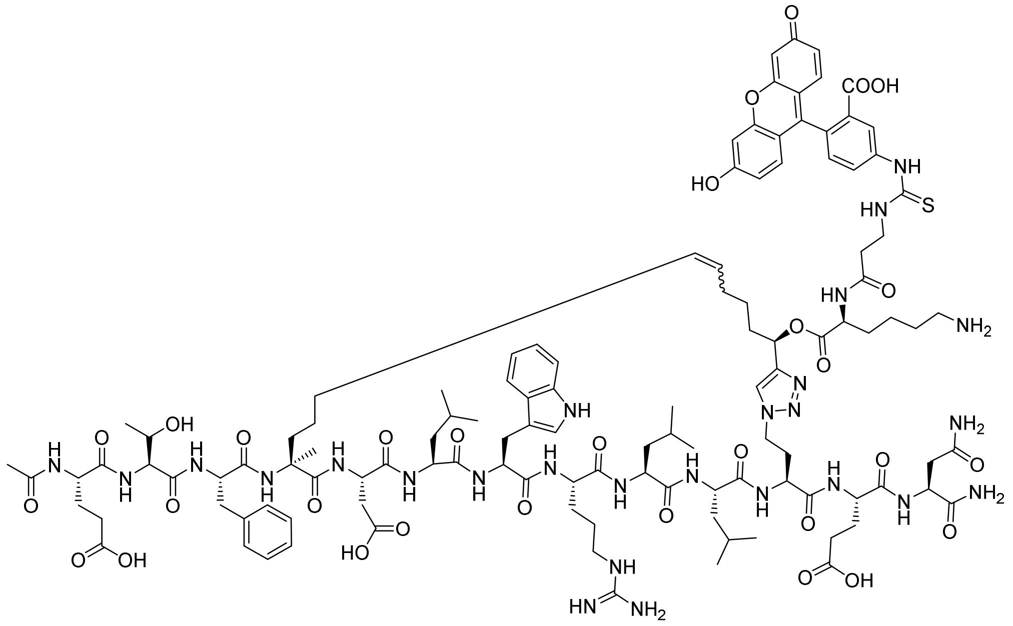

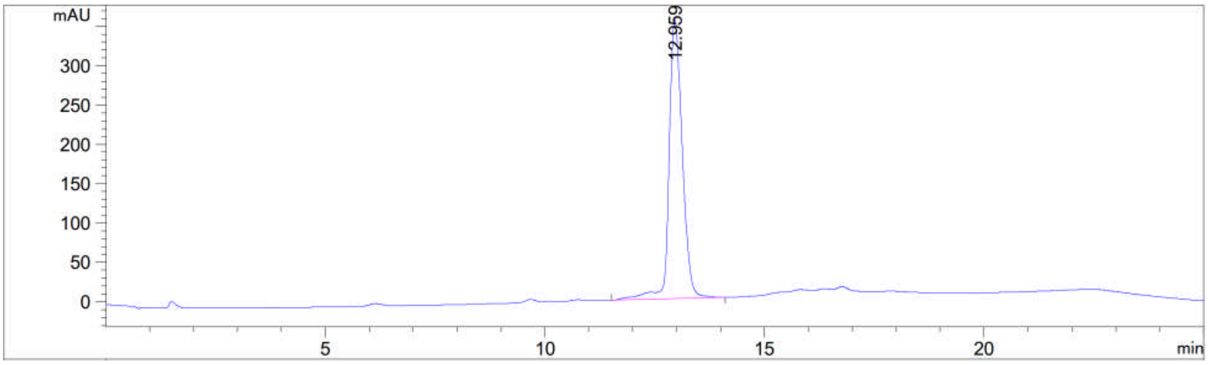

Area Percent Report:

Signal: 220.4 nm
Reference: 360.1

Peak #	Retention Time (min)	Width (min)	Area (mAu × s)	Height (mAu)	Area %
**1**	12.959	0.3231	7452.03174	354.65808	100.0000
**Totals:**			7452.03174	354.65808	

Peak Purity:

Purity factor: 998.495 (186 of 206 spectra exceed the calculated threshold limit.)

Threshold: 999.662

Reference: Peak start/end spectra (integrated) (11.519/14.106)

Spectra: 5

Noise Threshold: 0.064 (12 spectra)

5.2 Example HPLC Chromatographs of Crude SP1 Stapling Mixture

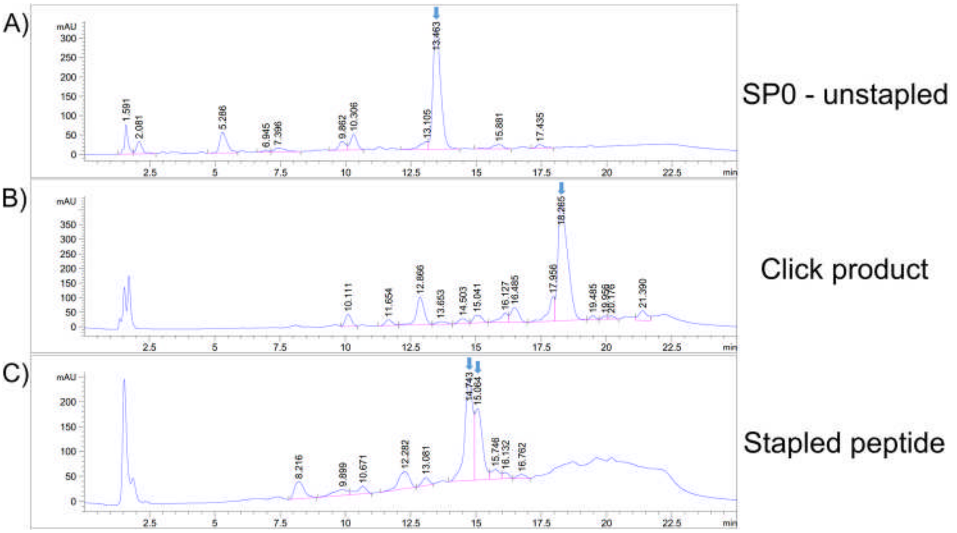

Figure S4. Crude HPLC chromatographs of A) **SP0**, reaction mixture after B) conjugating with Linker **1** using CuAAC conditions and C) stapling using RCM conditions. The blue arrows denote the product peptide peak/s.

5.3 Example HPLC Chromatographs of Crude SP1 Expansion Mixture

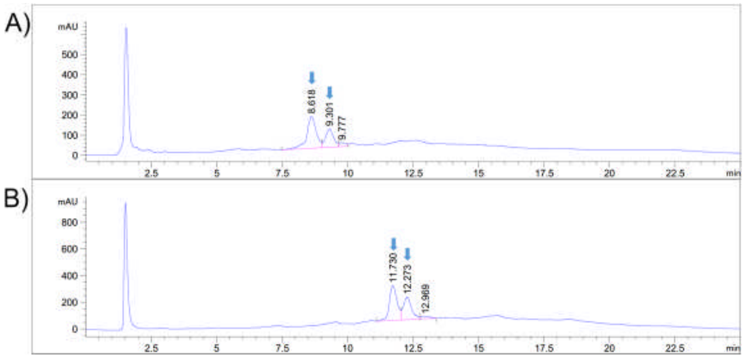

Figure S5. Crude HPLC chromatograph of A) **SP1-K** and B) **SP1-βA-F**. The blue arrows denote the product peptide peak.

5.3 Circular Dichroism

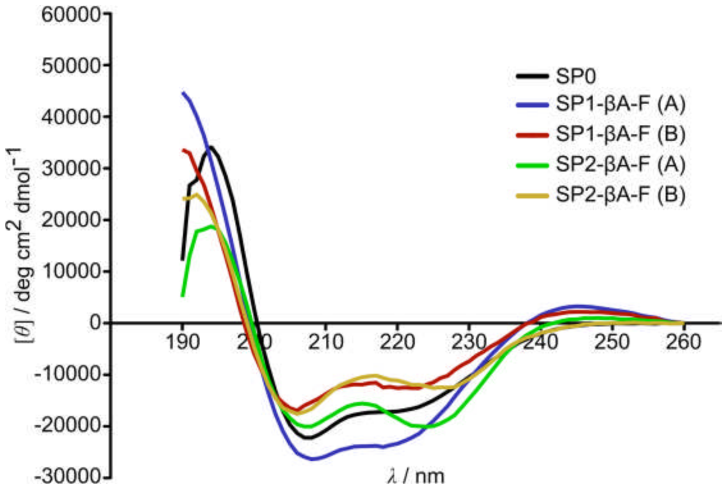

Figure S6. Circular dichroism spectra of peptides **SP1-βA-F (A/B) and SP2-βA-F (A/B).**

Table S4. Calculated helicity of unstapled and stapled peptides, based on mean residue ellipticity at 222 nm.

Peptide	Helicity (%)
**SP1-βA-F (A)**	68
**SP1-βA-F (B)**	34
**SP2-βA-F (A)**	64
**SP2-βA-F (B)**	37

6. References

[1] E. D. Goddard-Borger, R. V. Stick, *Org. Lett.* **2007**, *9*, 3797–3800.

[2] Y. Lau, D. R. Spring, *Synlett* **2011**, 1917–1919.

[3] F. R. Pinacho Crisóstomo, R. Carrillo, L. G. León, T. Martín, J. M. Padrón, V. S. Martín, *J. Org. Chem.* **2006**, *71*, 2339–2345.

[4] A. Rodríguez, M. Nomen, B. W. Spur, J.-J. Godfroid, T. H. Lee, *Eur. J. Org. Chem.* **2000**, *2000*, 2991–3000.

[5] Z.-X. Wang, *FEBS Lett.* **1995**, *360*, 111–114.

[6] Y. S. Chang, B. Graves, V. Guerlavais, C. Tovar, K. Packman, K.-H. To, K. A. Olson, K. Kesavan, P. Gangurde, A. Mukherjee, T. Baker, K. Darlak, C. Elkin, Z. Filipovic, F. Z. Qureshi, H. Cai, P. Berry, E. Feyfant, X. E. Shi, J. Horstick, et al., *Proc. Natl. Acad. Sci.* **2013**, *110*, E3445–E3454.
